# Myo-MOVES: a custom electrical stimulation system for functional studies of 3D bioengineered muscle

**DOI:** 10.1039/d5lc00614g

**Published:** 2025-10-01

**Authors:** Martín Ruiz-Gutiérrez, Ainoa Tejedera-Villafranca, Sergi Pujol-Pinto, Javier Ramón-Azcón, Juan M. Fernández-Costa

**Affiliations:** a Institute for Bioengineering of Catalonia (IBEC), The Barcelona Institute of Science and Technology (BIST) C/Baldiri Reixac 10-12 E08028 Barcelona Spain jramon@ibecabrcelona.eu jfernandez@ibecbarcelona.eu; b Programa de doctorat en Biomedicina, Universitat de Barcelona (UB) Spain; c Institució Catalana de Reserca i Estudis Avançats (ICREA) Passeig de Lluís Companys, 23 E08010 Barcelona Spain

## Abstract

Electrical pulse stimulation (EPS) is used to replicate motor neuron activation in muscle tissues, enabling *in vitro* studies of muscle contraction. However, both custom-built and commercial existing EPS systems often suffer from significant limitations, including limited scalability, high cost, and lack of flexibility for experimental adaptation. This work presents the Myo-MOVES platform, a practical solution for stimulating 3D skeletal muscle tissues. The device has been designed as an intuitive EPS system consisting of two main components: a selector and a stimulator that adapts to commercial 24-well culture plates. The Myo-MOVES selector enables targeted stimulation of single or multiple wells, while the stimulator delivers electrical signals *via* graphite electrodes to the plate containing 3D skeletal muscle samples. The Myo-MOVES platform was technically validated and employed as a proof of concept to investigate sarcolemmal damage induced by muscle contraction in Duchenne muscular dystrophy (DMD) 3D skeletal muscle tissues. Taking advantage of the versatility of the device, we validated Myo-MOVES through the assessment of force generation in DMD engineered muscle tissues and the detection of contraction-induced sarcolemmal damage *via* Evans blue dye uptake and the release of creatine kinase (CK), the gold standard marker of muscle damage. These findings demonstrate the feasibility of using Myo-MOVES to induce and study functionally relevant disease phenotypes in DMD 3D skeletal muscle tissues. These results highlight the system's potential as a valuable tool for future applications in the field of 3D skeletal muscle tissue engineering, including drug screening and the study of DMD therapies and other muscular diseases.

## Abstract 2

Myo-MOVES is a versatile platform for electrical pulse stimulation (EPS) that mimics motor neuron activation in muscle tissue and enables a wide range of *in vitro* muscle contraction studies.

## Graphical abstract

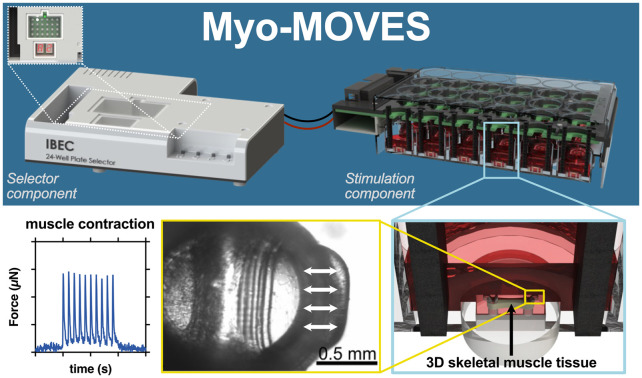

## Introduction

Skeletal muscle, the most abundant organ of the human body,^1^ plays a crucial role in many essential functions, including the locomotive and respiratory system and metabolism.^2–5^ Alongside cardiac muscle, it falls within the subtype of striated muscle, and it is characterised by its innervation voluntary movement, high energy requirement, susceptibility to fatigue, and highly organised structure.^6^ At the cellular level, myoblasts serve as the fundamental building blocks of muscle tissue. These precursor cells differentiate into myocytes, which further fuse into myotubes. Myotubes are enclosed within the sarcoplasm, which is wrapped by the sarcolemma, forming muscle fibres.^7^

Muscle can be affected by several pathologies of diverse origins. One notable example is Duchenne muscular dystrophy (DMD), a rare genetic disorder with no cure which results in devastating damage and loss of skeletal muscle, known as atrophy.^8,9^ Autoimmune conditions such as myasthenia gravis,^10^ infections,^11,12^ metabolic disorders,^13,14^ exposure to toxic substances or drugs^15,16^ and endocrine imbalances^17,18^ also contribute to muscle dysfunction. Even the natural ageing process, in the form of sarcopenia,^19^ results in the gradual decline of muscle mass and strength, highlighting the vulnerability of this vital organ.

Despite the different mechanisms of each muscular disease and their multiple secondary manifestations, they typically exhibit progressive muscle waste and weakness. Therefore, the development of *in vitro* functional muscle cell culture capable of contracting is essential to accurately replicate *in vivo* characteristics of skeletal muscle tissue.^20^ Traditional two-dimensional (2D) cell cultures offer simplicity and low cost as their main advantages. However, they fail to fully mimic *in vivo* muscle properties. The lack of three-dimensional (3D) structures disrupts cell–cell and cell–extracellular matrix interactions, leading to altered signalling pathways, impaired function, and ultimately, loss of key muscle phenotypes.^21^ To address these limitations, advanced 3D skeletal muscle models have been developed, enabling functional studies by measuring the force generated during contraction, thus providing a more physiologically relevant platform for research.^22–33^

Typically, 3D skeletal muscle models are fabricated using casting moulds consisting of a pool containing two protruding pillars. Cells are encapsulated within these casting moulds in a hydrogel. Uniaxial tension mechanical cues from the pillars promote alignment and differentiation of the myotubes as the hydrogel compacts.^34,35^ Other approaches incorporate 2D techniques on advanced substrates, such as cantilevers where muscle contraction results in the bending of the cantilever.^36,37^ Additionally, bioprinting enables the fabrication of more intricate structures by precise deposition of bioinks containing muscle cells into predefined architectures, offering greater control over tissue organisation and functionality.^38,39^

Muscle contraction is triggered by motoneurons that induce changes in the transmembrane potential of myotubes, a process driven by the controlled modulation of membrane permeability to diffusible ions. To replicate this process *in vitro*, electrical pulse stimulation (EPS) is commonly used to induce contraction. EPS involves applying electrical fields to muscle cultures to trigger contraction. Different contraction types, such as twitch and tetanic contraction, can be induced by modulating the frequency of the pulses.^40^ Precise control of stimulation parameters is particularly valuable for muscular damage research, as it can provide insights into exercise-induced stress patterns on the muscles.^41^ This technique is also beneficial in drug testing, as it enhances the contrast between damage observed in healthy control and disease cell lines, allowing for clearer observation of therapeutic effects and enabling statistically significant conclusions from experimental data. EPS has been used to study sarcomere formation and hypertrophic responses, metabolic and inflammatory responses related to obesity and diabetes, and contraction-induced myokines, among others.^42^ By enhancing the physiological relevance of *in vitro* muscle models, EPS has become an essential tool for advancing contraction-related research.

EPS experiments require a reliable system to deliver electrical pulses to the muscle tissues. Basic setups consist of a Petri dish with culture medium containing a muscle sample along with two electrodes, connected to a wave generator.^43,44^ While simple, these setups lack reproducibility and scalability. More advanced systems use a stimulation plate, which mainly consists of a printed circuit board (PCB) with electrode pairs aligned with the wells of commercial culture plates. These plates replace the standard lid and allow EPS application to both 2D and 3D cultures. Some commercial stimulation plates are available,^45^ but many laboratories design custom setups tailored to their specific research needs. A key limitation of commercial stimulation plates is the inability to selectively stimulate individual wells, as they apply the same electrical regimen to all samples in the plate at once. This poses challenges for functional experiments that rely on video microscopy-based measurement of pillar deflection to analyse contraction. Since only one sample can typically be recorded with sufficient resolution at a time, repeated stimulation of all tissues in the plate can lead to muscle fatigue, altering contractile behaviour and compromising experimental results. Some more advanced commercial systems integrate the generation of 3D tissues, the functional experimentation, and data collection and processing. Despite their practicality, the high initial cost of these systems and their consumables prevents most research laboratories from using them.^46–48^

To address these limitations, in this work, we developed the Myo-MOVES platform, a novel electrical stimulation system for the functional study of 3D skeletal muscle tissues. Our system consists of two parts: (1) a selector that allows researchers to choose which samples to stimulate, enabling targeted EPS application, and (2) a custom stimulation plate designed for 24-well culture plates. This setup allows for the implementation of multiple stimulation modes within a single experiment, significantly improving experimental flexibility and scalability. As a proof of concept of the utility of Myo-MOVES, we applied this novel setup to explore and screen the functional induction of relevant disease phenotypes of DMD. Specifically, we validated the system by assessing force generation and detecting contraction-induced sarcolemmal damage *via* Evans blue dye (EBD) uptake and release of creatine kinase (CK), the gold standard marker of muscular damage. Myo-MOVES significantly improved throughput and experimental versatility, while also reduced researcher workload and minimised the risk of sample contamination and damage. By streamlining the EPS process and enhancing experimental control, our setup represents a valuable advancement in functional skeletal muscle research.

## Materials and methods

### PCB design and fabrication

Electric schematics and PCB designs were made using KiCad EDA 6.0. The connections between components were automatically generated using FreeRouting.^49^ Printed circuit boards were fabricated by Circuitos Impresos 2CI, S.L. (Barcelona, Spain).

### Selector design

The electric schematic of the selector (SI, A0.1 and A0.2) was divided into two main parts: (1) an array of ten relays, with flyback diodes and controlled by the activation of optocouplers, and (2) a dashboard consisting of two 7-segment displays and 24 white LEDs, each representing a well of the culture plate. The device was powered at 12 V. The stimulation signal was received through a BNC connector or a 10-way, 2 rows IDC male connector, while the branched signal was sent through another 10-way IDC connector. The selector was controlled by an Arduino Mega 2560 Rev3, with four pushbuttons to operate the system. The Arduino board was programmed using Arduino IDE.

### Stimulator design

A plated through-hole footprint was designed to securely house graphite electrodes, maintaining a separation distance of 10 mm between their inner surfaces (SI, A1.2). Each pair of electrodes, designated for a specific well, was connected: the first electrode to a designated row and the second electrode to a corresponding column through a Schottky diode. To receive the branched stimulation signals, a 10-pin IDC header was incorporated, while a 24-pin IDC connector was implemented to measure the voltage drop within the well, excluding the diode's influence (SI, A1.1).

### 3D design and printing

SOLIDWORKS (Dassault Systèmes SolidWorks Corp.) was used to design 3D models of a box for the selector (SI, B0–B6) and a support for the stimulator (SI, C0–C7). Models were exported as Standard for the Exchange of Product model data files (STEP) and sliced using Bambu Studio software (Bambulab GmbH). They were then printed using the Bambu Lab X1-Carbon (Bambulab GmbH) fused deposition modelling 3D printer. The box of the selector was printed using PLA (polylactic acid, Bambulab PLA basic) for the base and the lid, transparent PETG (polyethylene terephthalate glycol, OVERTURE PETG transparent) for the dashboard windows, and TPU (thermoplastic polyurethane, Creality HP-TPU) for the pads. The material used for the support of the stimulator was ASA (acrylonitrile styrene acrylate, Bambulab ASA) due to its high resistance to UV radiation.

### Platform assembly

The components of the selector were manually welded using a Weller WE1010 soldering station and a Weller WEP 70 soldering iron. The electrodes for the stimulator were cut manually from high purity graphite plates of 100 × 100 × 2 mm (Micro to Nano, Haarlem, The Netherlands) into smaller pieces of 25 × 5 × 2 mm. 3D designed and printed tools were used to make notches in the graphite plates using a cutter (SI, D0 and D1). Then, another tool was used to break the graphite taking advantage of its brittleness (SI, D2). The electrodes were mounted on the PCB and aligned using another 3D printed tool (SI, D3). They were glued to the board using MG Chemicals 8331D silver conductive epoxy adhesive, due to the non-adherence of tin to graphite. The SMD diodes were welded using the DURATOOL D01841 hot air rework station and MG Chemicals 4902P-15G solder paste. The selector PCB was secured to the base of the 3D printing box using four M3 screws and the lid was attached using four M4 screws. The stimulator PCB was attached to its support using six M3 stainless steel screws.

### Cell culture

Immortalised human muscle precursor cells from a healthy control (control 2, clone E4) and a DMD patient (DMD 2, clone G82) were generously provided by Dr Bénédicte Chazaud (Institut NeuroMyoGène, Lyon, France).^50^ Cells were cultured in growth medium consisting of skeletal muscle basal medium (C-23060, PromoCell GmbH), skeletal muscle supplemental mix (C-39365, PromoCell GmbH), 10% fetal bovine serum (FBS) (10270-106, Gibco™) and 100 U mL^−1^ penicillin and 100 μg mL^−1^ streptomycin (15140-122, Gibco™). Cell cultures were maintained at 37 °C in a humidified atmosphere with 5% CO_2_.

### Fabrication of 3D skeletal muscle tissues

Human 3D skeletal muscle tissues were fabricated as previously described.^34^ Briefly, cells were encapsulated at 2.5 × 10^7^ cells per mL in a cell/hydrogel mixture seeded on polydimethylsiloxane (PDMS; Sylgard 184 silicone elastomer kit, Dow Corning) casting moulds with two vertical flexible pillars.^3^ The hydrogel mixture consists of 30% v/v Corning® Matrigel® GFR Basement Membrane Matrix (Corning®), 2 units per mL of thrombin from human plasma (Sigma-Aldrich) and 4 mg mL^−1^ of fibrinogen from human plasma (50% v/v) (Sigma-Aldrich). Tissues were kept in growth media containing 1 mg mL^−1^ of 6-aminocaproic acid (ACA; Sigma-Aldrich) for two days and then switched to differentiation medium (97% DMEM, high glucose content, with GlutaMAX™ supplement, 61965-026, Gibco™; 1% KnockOut™ serum replacement, 10828-028, Gibco™; 1% insulin–transferrin–selenium–ethanolamine (ITS-X), 51500-056, Gibco™ and 1% penicillin–streptomycin–glutamine (PSG), 10378-016, Gibco™) with ACA for seven days. Half of the volume was replaced every 48 hours.

### Electrical pulse stimulation (EPS)

The stimulator was cleaned before every EPS experiment. The electrodes were submerged in 70% etOH under agitation for a day, followed by another day of Milli-Q® water submersion. This allowed the release of absorbed molecules from the porous surface of the graphite. Before the experiment, the electrodes were sterilised by 10 minutes of UV light exposure. Samples were placed in a 24-well plate with fresh differentiation medium before stimulation. They were stimulated under different regimens of varying frequency and total stimulation time. A wave generator (NF WF1974, NF Corporation) was used to produce the stimulation signal, which was amplified 10× using a custom amplifier. The signal used was a monophasic square wave of 10 V_p_ with a 50% offset and 1 ms wide pulses. The reproducibility of the signal applied in each well was tested using a Siglent SDS 1104X-E oscilloscope to record the waveform at 1, 20 and 150 Hz in a 24-well plate filled with 1 ml of differentiation medium per well (SI Fig. S1–S4). After the EPS, the samples were left to rest for one hour, and then the supernatant was collected. The stimulator was placed inside an XL S1 cell incubator maintaining physiological conditions (37 °C and 5% CO_2_) during the EPS. To compare with a conventional system, tissues were stimulated using a standard 12-well plate with two graphite electrodes embedded in the lid, previously described and published,^22,23^ and a commercial stimulation plate (IonOptix 6-wells C-Dish^51^). Stimulation was applied using the same waveform parameters. Brightfield images and recordings of the contracting 3D tissues were taken inside the stimulator using a Zeiss Axio Observer Z1/7. The recordings of the movement of the PDMS pillars were analysed as previously described to obtain the force that the 3D skeletal muscle tissue performed.^22^ Evans blue dye (EBD) muscle damage assay was performed as previously described.^22^

### Enzyme-linked immunosorbent assay (ELISA)

Creatine kinase (CK) levels in supernatants were measured using a sandwich ELISA protocol adapted for 96-well plates. Plates were coated overnight at 4 °C with 50 μL per well of capture antibody at 0.625 μg mL^−1^ in coating buffer (15 mM Na_2_CO_3_, 35 mM NaHCO_3_, pH adjusted to 9.6 using HCl and NaOH), sealed with adhesive plate sealers. The next day, the plates were washed with PBST (PBS with 0.05% Tween-20) using an automated plate washer. Wells were blocked with 200 μL per well of 1% BSA in PBS for 2 hours at room temperature (RT), then emptied and dried gently with absorbent paper. Standard curves were prepared using CK concentrations ranging from 1500 ng mL^−1^ to 1.15 ng mL^−1^, diluted in differentiation medium. All samples and points of the calibration curve were tested with two technical replicas. Samples and standards (100 μL per well) were loaded into the plate and incubated for 1 hour at RT. After washing, 50 μL per well of biotinylated detection antibody (1.25 μg mL^−1^ in 1% BSA-PBST) was added and incubated for 1 hour at RT. Following another wash step, 50 μL per well of streptavidin-poly-HRP (0.1 μg mL^−1^, diluted in 1% BSA-PBST) was added and incubated for 15 minutes. After a final wash, wells were incubated in the dark with 100 μL per well of substrate solution containing citrate buffer (40 mM sodium citrate tribasic dihydrate, Sigma-Aldrich, pH adjusted to 5.5 using acetic acid), TMB (6 mg mL^−1^ in DMSO), and 1% H_2_O_2_ for 30 minutes or until colour developed. The reaction was stopped by adding 50 μL per well of 2 N H_2_SO_4_, and absorbance was read at 450 nm using a spectrophotometer (Gen4 software).

### Immunohistochemistry and confocal imaging

Immunohistochemistry procedures followed the previously published protocol.^22^ Cryosections or whole-mount samples were permeabilised with PBS-T (0.1% Triton-X in PBS) for 10 or 15 minutes, and then blocked with a buffer containing 0.3% Triton-X and 3% donkey serum in PBS for 30 minutes or 2 hours at room temperature. Subsequently, samples were incubated with the primary antibody (1 : 200, monoclonal mouse anti-human α-actinin (sarcomeric), Merck Life Science #A7811) in blocking buffer overnight at 4 °C. Following three 5-minute washes with PBS-T, samples were incubated for 45 minutes or 2 hours at room temperature with either fluorophore-conjugated secondary antibody (1 : 200, polyclonal donkey anti-mouse IgG, Alexa Fluor™ 488-conjugated secondary antibody, Invitrogen #A21202) or phalloidin (1 : 400, Alexa Fluor™ 594 phalloidin, Invitrogen #A12381) to stain F-actin. After another three 5-minute washes with PBS-T, whole-mount samples were stained with DAPI (4′,6-diamidino-2-phenylindole, Life Technologies) for 30 minutes. Samples were rinsed with PBS for 5 minutes before mounting with either VECTASHIELD Plus Mounting Medium with DAPI (Palex) or Fluoromount-G™ Mounting Medium (Thermo Fisher). Confocal fluorescence images were taken with a ZEISS LSM800 confocal laser scanning microscope. Images were analysed using the Fiji image processing package.^52^

### Statistical analysis

For the analysis of ELISA data, a standard curve was generated using known concentrations of the analyte. The standard curve was fitted using a four-parameter logistic (4PL) model using Prism 8 software (GraphPad). The limit of detection was defined as the concentration corresponding to the intersection of the lower asymptote of the four-parameter model and the 10% confidence interval. The comparisons between groups were performed using Prism 8 software (GraphPad). Statistical test outcomes are reported as mean ± SD. Pairs of samples were compared using a two-tailed Student *t*-test (*α* = 0.05). Differences between groups were considered significant when *P* < 0.05 (**P < 0.01).

## Results and discussion

### General overview of the custom-built stimulation setup for functional testing of 3D skeletal muscle tissues

**Fig. 1:**
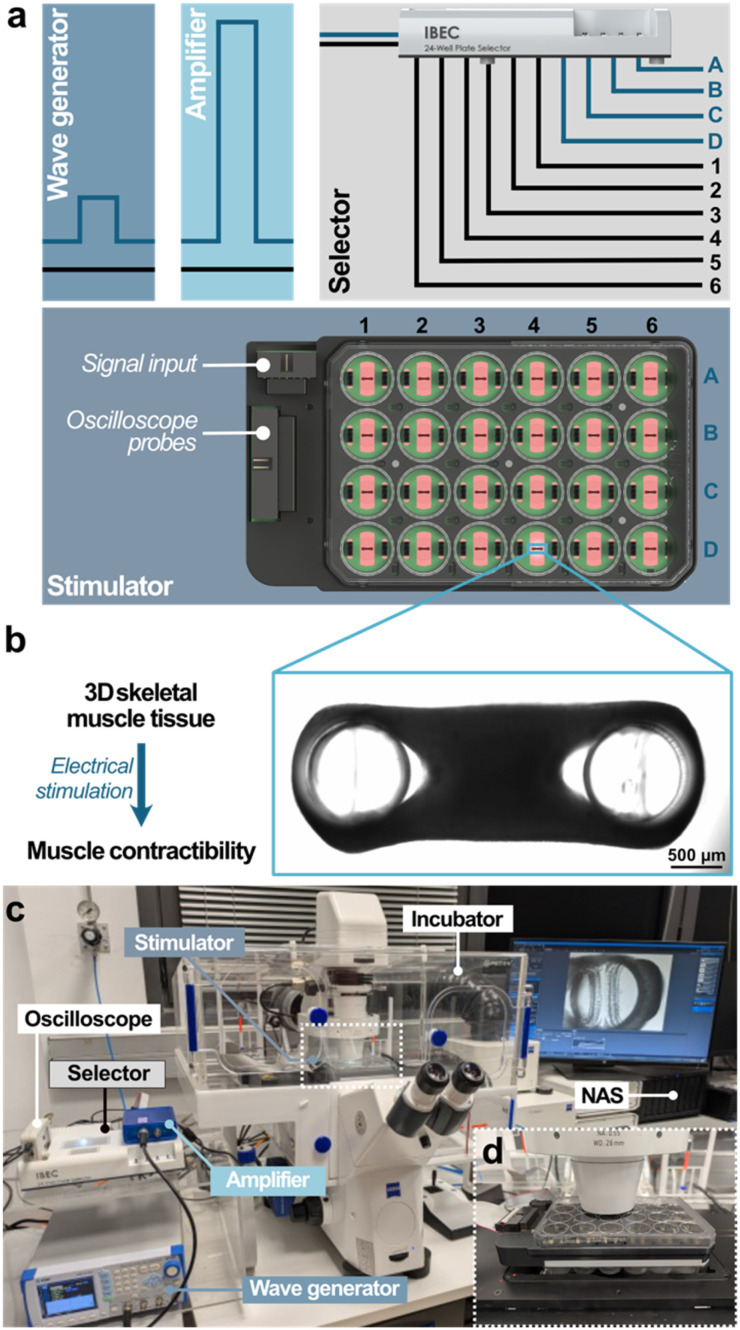
General diagram of Myo-MOVES. (a) Schematic of signal delivery: a wave generator connected to a 10× amplifier produces the stimulation signal. A selector splits this signal into four phase channels (A–D) and six ground channels (1–6), which can be individually connected or disconnected *via* relays. The stimulator directs the signal to samples arranged in a matrix-like configuration. (b) Brightfield microscopy image of a 3D bioengineered skeletal muscle tissue. (c) Photograph of the experimental setup. The electric pulse stimulation system is integrated with a microscope and an incubator, allowing real-time visualisation of muscle contractions. Data are recorded and stored on a network-attached storage (NAS) device. (d) Close-up image of the stimulator component.

To develop a versatile, affordable, and user-friendly system for the electrical stimulation of 3D bioengineered skeletal muscle tissues, we designed and built the Myo-MOVES (MOdular Versatile Electrical Stimulation) platform. Our goal was to create an in-house, modular, and customisable system that could easily adapt to a wide range of experimental needs. To achieve this, we focused on the following key features: (1) a large enough capacity to accommodate multiple samples simultaneously; (2) the ability to selectively stimulate individual wells without affecting neighbouring samples, enabling the recording of single samples without fatiguing the rest; (3) the flexibility to stimulate multiple wells in parallel for experiments that assess parameters other than contractile force; and (4) compatibility with standard commercial culture plates and optical accessibility for real-time observation under a microscope. In line with these requirements, our Myo-MOVES platform consists of two parts: the selector, which is used to designate the wells to be stimulated, and the stimulator, which delivers the signal to the tissues (Fig. 1). The stimulation signal is provided by a wave generator and amplified 10 times. The stimulation signal arriving to the selector is branched and sent to the stimulator through a 10-way cable after passing through 10 relays. Four ways send signal, while the other six are connected to the ground reference. This allows the establishment of a matrix organisation on the commercial 24-well plate, which consists of 4 rows and 6 columns (Fig. 1a). By connecting each row to one way of stimulation signal and each column to a ground reference, the operation of the relays permits the delivery of current to a single, a selection, or all wells at once. An oscilloscope is used to monitor the signal arriving to the tissues. During stimulation, tissue contraction was recorded using a microscope (Fig. 1b). The stimulation protocol of the wave generator was controlled by a computer, and the recorded data were stored in a network attached storage (NAS) (Fig. 1c).

### Design and functionality of the Myo-MOVES selector module

**Fig. 2:**
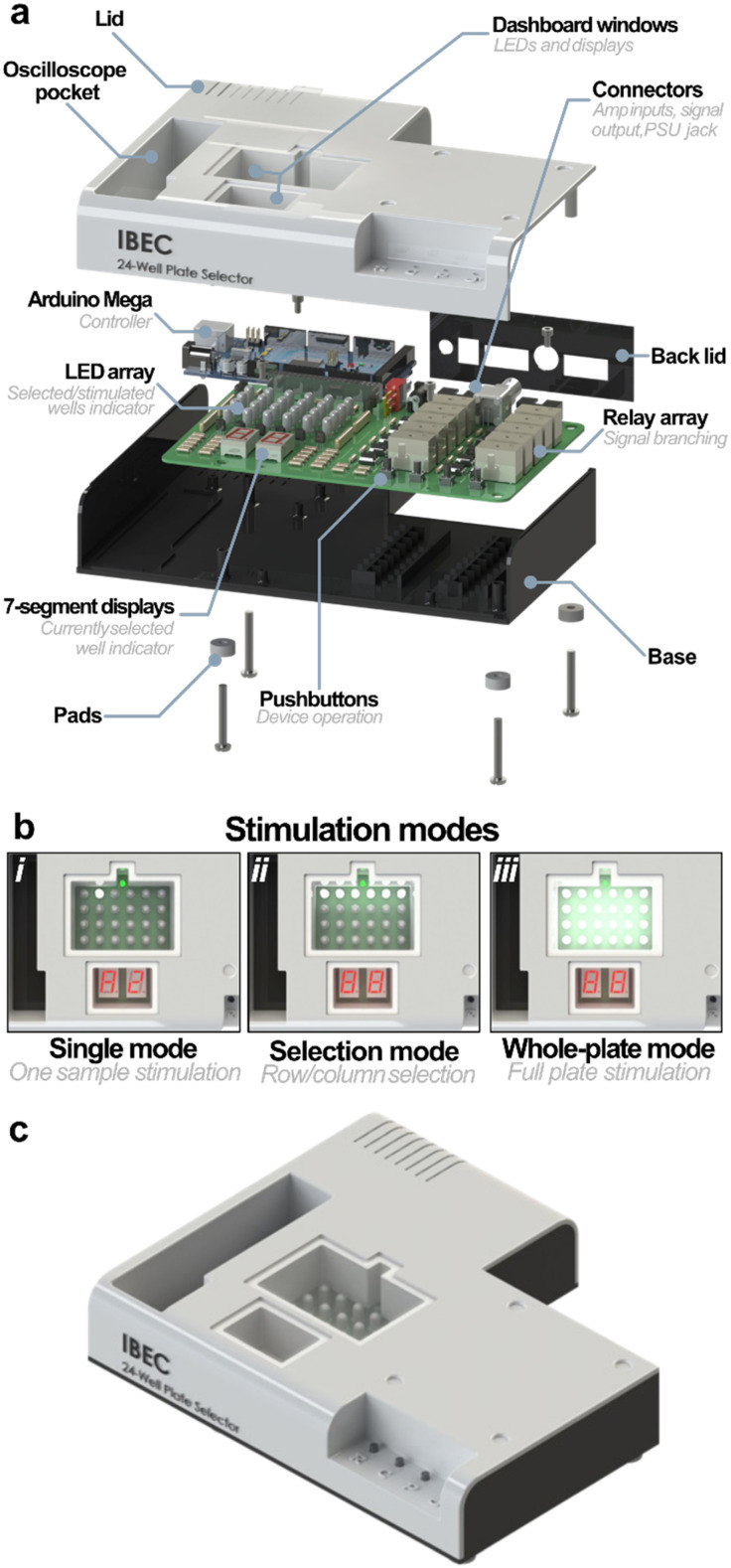
Detailed rendered views of the selector. (a) Exploded view of the selector, illustrating its internal components and assembly. (b) Top view of the control dashboard, showcasing the interface under three different stimulation modes. (c) Isometric view of the stimulator, highlighting its overall design and layout.

The selector (Fig. 2c) consists of a PCB with a relay array for branching of the stimulation signal and a dashboard with LEDs and 7-segment displays. It is enclosed in a 3D printed box together with its controller, an Arduino Mega (Fig. 2a). To achieve maximum versatility, the controller was programmed to allow three stimulation modes (single, selection and whole-plate) designed for different types of experiments (Fig. 2b). Four pushbuttons are used to control the device, and the dashboard provides information to the user to better operate the device.

The first mode, single mode (Fig. 2bi), is used to stimulate one sample at a time. The system allowed toggling the selected well by using the first two pushbuttons, selecting a row and column. Each successive press changes the value of the selected row (A–D) or column (1–6) in a loop. The current selection is shown by the two 7-segment displays. The third button connects the corresponding relays to deliver the stimulation signal to the chosen well of the culture plate. The dashboard LED representing the selected well lights up once the relays are activated. This mode was made for experiments which require the recording of the contracting tissue, such as force-measuring experiments. By using this mode, up to 24 samples can be loaded into the culture plate in a single go, and then they can be stimulated and recorded one at a time. Simultaneous sample loading presents several advantages over other systems such as simple pairs of electrodes or plates without the possibility of sample selection. The time spent loading the samples is dramatically reduced, since the user does not have to change the sample after every stimulation. This, in turn, reduces the risk of contamination and exposure to non-sterile environments.

The second mode, selection mode (Fig. 2bii), allows the stimulation of several wells at once. In this case, the user can choose between rows and columns and add them to the selection using the fourth pushbutton. The selection of rows or columns is toggled by pressing their corresponding button. Once one is chosen, its display lights up, while the other dims. When a well will be stimulated due to the selection of its row and column at once, its representing LED turns on. When connecting the relays, both the selected LEDs and displays blink.

This mode was intended to be used when different stimulation regimens want to be tested on several biological replicas. For instance, the plate can be fully loaded with samples, evenly divided between control and diseased groups. Then, a stimulation regimen can be applied to each row, having a total of four regimens applied to 3 replicas of control and diseased samples. At the end of the stimulation, supernatants can be collected for their use in other assays.

The main limitation of this mode is the inability to work with several signals. Only one signal can be received and branched at a time, meaning that for the application of different stimulation regimens, selections of samples must be stimulated sequentially, changing the settings of the wave generator between batches. Furthermore, because of the matrix-like connection of the wells, certain selections, such as diagonal lines of wells, are not possible.

Finally, the whole-plate stimulation mode (Fig. 2biii) activates all relays at once. This mode was intended to easily stimulate the full plate, achieving the maximum number of replicas for a given EPS protocol. In all cases, the displays blink when the relays are activated, and the selection of different wells is locked until the active relays have been disconnected.

The box of the selector ensures electrical safety by enclosing all electronic components. It consists of a base with threaded supports for the PCB and the Arduino and a lid with holes aligned with the LED array and 7-segment displays, as well as a pocket to store a portable oscilloscope. The dashboard is covered by transparent 3D printed windows. A 3D printed plate with holes is used to cover the back of the device while maintaining access to the connectors. The box is closed using pairs of flaps and slots and four screws, which also hold the pads in place.

The stimulation signal coming from the amplifier can be received *via* a BNC connector, for commercial amplifiers, or through a 10-way connector, adapted for a custom amplifier. After going through the relay array, the signal is outputted though another 10-way port.

### Stimulator design for targeted EPS in standard well plates

**Fig. 3:**
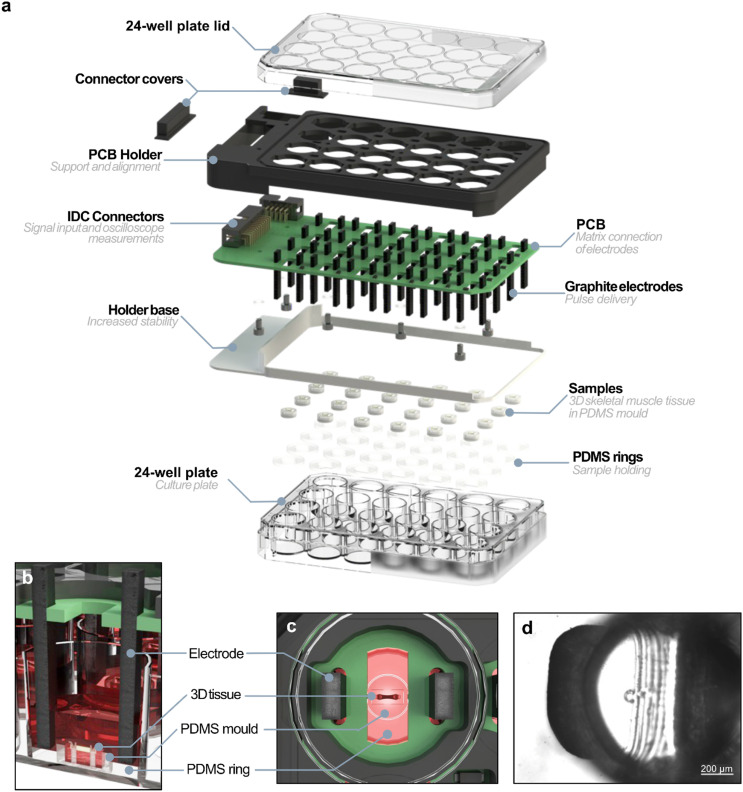
Detailed rendered views of the stimulator and sample setup. (a) Exploded view of the stimulator, showing its internal structure and component arrangement. (b) Cross-sectional view and (c) top view of a well containing a 3D skeletal muscle sample in differentiation medium. (d) Brightfield microscopy image of a PDMS pillar within the mould, used to monitor deflection and calculate the contractile force generated by the tissue.

The stimulator was designed to be compatible with a commercial culture plate (Thermo Fisher Nunc) to make it easier to use. It receives the branched stimulation signal from the selector and delivers it to the samples according to the chosen selection. A 3D designed and printed support is used to adjust the stimulator PCB on top of the commercial culture plate, enclosing and ensuring sterile conditions (Fig. 3a). The cell culture plate lid is fitted on the upper part while the lower part of the support fits the culture plate itself to enclose the device. The pins of the connector are covered to restrict access to conductive parts. The protruding distance of the electrodes was adjusted so that they reach as deep as possible in the wells without touching the bottom, ensuring electric field in the sample (Fig. 3b). PDMS rings are used to hold the moulds containing the samples in the centre of the wells. These rings were glued to the bottom of the culture plate.

The PCB of the stimulator was designed so the electrodes were aligned with the wells of the commercial culture plate. It has holes between the electrode holders to allow microscopy imaging while conducting experiments (Fig. 3c and d). The diodes prevent undesired current flow in unselected wells, and the 24-pin connector can be used together with the 10-pin connector to measure the voltage drop directly in the well, excluding the diode. We generated a 10 V_P_ square signal with 1 ms long pulses at 1, 20 and 150 Hz, and measured the actual waveform arriving to all wells of a plate (SI Fig. S1(a and b)–S4) using an oscilloscope. We found mean peak voltage values of 10.00 V ± 0.04 V, 10.00 V ± 0.03 V and 10.02 V ± 0.03 V for each respective frequency. We also measured the voltage drop in the diode and found it to be 0.38 V ± 0.01 V across all frequencies. Subtracting this value from the average well potential allows us to determine the actual voltage drop in each well (9.62 V). The observed value confirms that the system delivers pulses close to the intended amplitude with high stability and reproducibility.

To load the samples in the plate, the whole PCB assembly can be easily removed, leaving only the base of the support in the original place of the commercial culture plate lid. The PDMS moulds containing the samples can then be placed in the centre of the PDMS rings using tweezers. Finally, the stimulator can be closed by simply sliding the support on top. This procedure can be done inside a biosafety cabinet if the 3D tissues need to be preserved after the EPS experiment. Sample contamination can be avoided as long as the proper cleaning and sterilisation protocol is followed.

To experimentally validate that our system operates as intended and delivers effective electrical stimulation comparable to conventional approaches, we compared the voltage within the wells of our platform to two reference systems: (i) a simple, custom-built setup consisting of a 12-well plate with electrodes embedded in the lid,^22,23^ and (ii) the commercially available C-dish.^51^ In both reference systems, we obtained voltage values similar to those measured in our platform (SI Fig. S1(c and d)), confirming that the electrical output in each well is within the expected range. Moreover, stimulation of muscle tissues cultured in our system resulted in contractile responses consistent with those observed using the conventional custom system (SI Fig. S5), with minor differences attributed to biological variability. This demonstrates that the electrical stimulation delivered by our device is functionally equivalent to conventional systems.

The Myo-MOVES platform presents several advantages over other commercial alternatives. First, the ability to load a notable number of samples and stimulate only the selected ones is a highly valuable feature for EPS experiments that not all platforms present. Furthermore, the simplicity of the stimulator makes it highly versatile. Other setups integrate muscle culture, stimulation and analysis. This makes work straightforward but also narrows the applicability and makes the user dependent on externally designed parts. In the case of our stimulator, the only constraint for the muscle culture sample is the space between electrodes. 3D bioengineered tissues of varying sizes encapsulated in any type of mould and even 2D cultures can be stimulated. The simple fabrication methodology of the stimulator makes it easy to assemble in-house and maintain reduced production costs, with material costs of around 200€ for the developed parts of the setup as of the writing of this article (SI Table S1). This includes all essential components required for assembly and operation. While exact pricing for commercial electrical stimulation systems varies and is often not publicly disclosed, the cost of the Myo-MOVES system is substantially lower than that of most commercially available multi-well ES platforms. This cost-effectiveness, combined with the system's flexibility and ease of assembly, makes Myo-MOVES a practical alternative for laboratories with budget constraints or those requiring customizable stimulation protocols.

### Functional characterization of Myo-MOVES across key use cases in DMD 3D muscle tissues

**Fig. 4:**
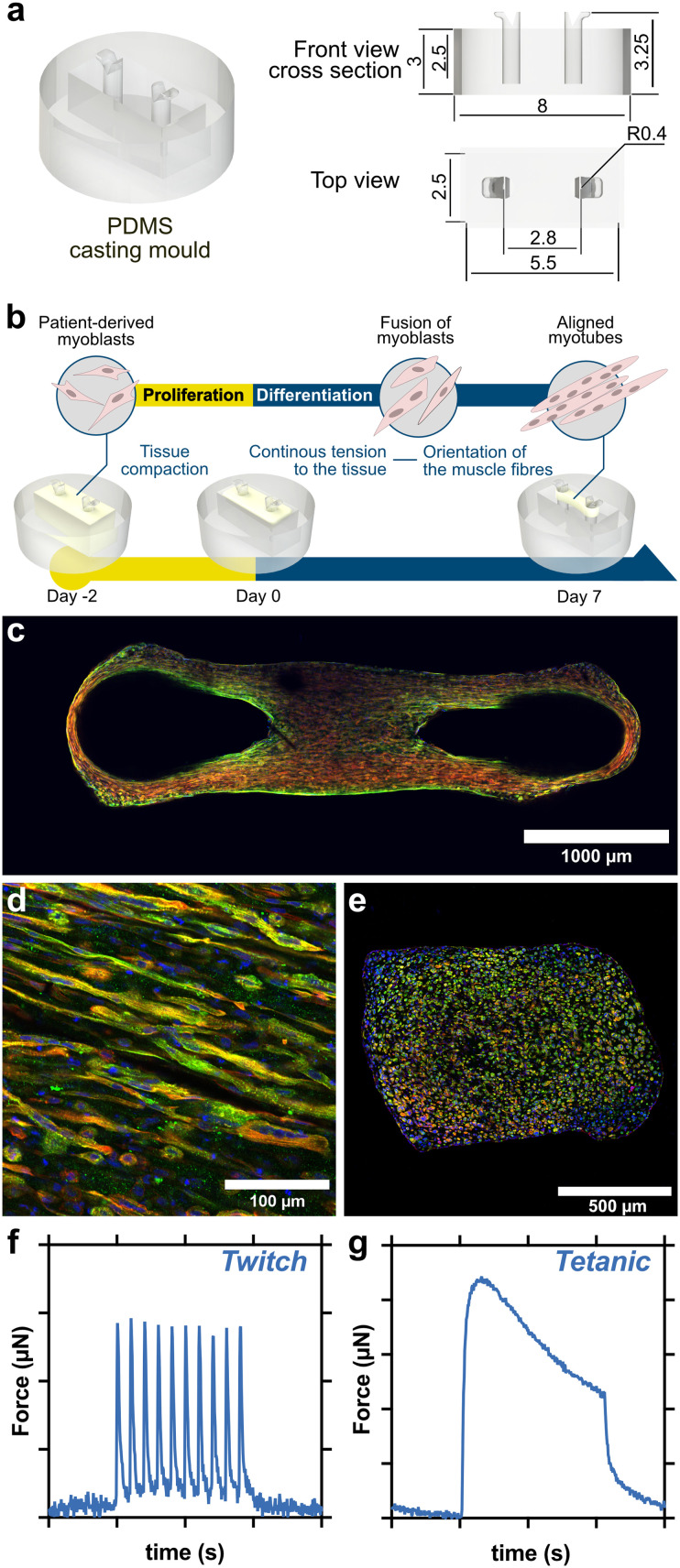
3D skeletal muscle tissue fabrication. (a) Schematic drawing of the PDMS mould. (b) Schematic representation of the fabrication process and experimental timeline. (c–e) Representative confocal images of a human 3D skeletal muscle tissue after seven days of differentiation stained for sarcomeric α-actinin (SAA, green), F-actin (red) and nuclei (blue). (b) Whole-mount views of the tissue. Scale bar = 1000 μm. (c) Higher magnification views of selected regions. Scale bar = 100 μm. (d) Transverse cross-sections of the tissue. Scale bar = 500 μm. (f and g) Representative twitch and tetanic contractions. Each division along the *x*-axis corresponds to 5 seconds. The *y*-axis scale varies between samples depending on tissue strength, with maximum forces typically ranging from 10 μN to 600 μN.

3D skeletal muscle tissues are a valuable tool for disease modeling, offering a more physiologically relevant environment than traditional 2D cultures.^24^ However, when the phenotypes of interest are linked to contractile function, as in the case of membrane fragility in DMD, EPS protocols must be specifically adapted to the engineered biological system. To address this need, the Myo-MOVES platform enables precise and customizable EPS delivery, supporting functional studies in diverse 3D muscle tissue configurations. We explored the different functional modes of the Myo-MOVES platform in a 3D skeletal muscle model of DMD, including the monitoring of contractile performance at various stages of differentiation and the assessment of membrane fragility using complementary approaches. To do this, we fabricated functional 3D skeletal muscle tissues using both healthy control (CNT) and DMD patient-derived myoblasts. Briefly, we used PDMS moulds consisting of a central well with two vertical posts, into which we added muscle progenitor cells embedded in a fibrin and Matrigel® matrix (Fig. 4a and b). During the first few hours after encapsulation, the hydrogel compacted, and the tension exerted by the posts on the hanging hydrogel facilitated the formation of aligned, multinucleated myotubes after 7 days of differentiation, as demonstrated by immunostaining for muscle-specific markers such as sarcomeric α-actinin (Fig. 4c–e, SI Fig. S6).

**Fig. 5:**
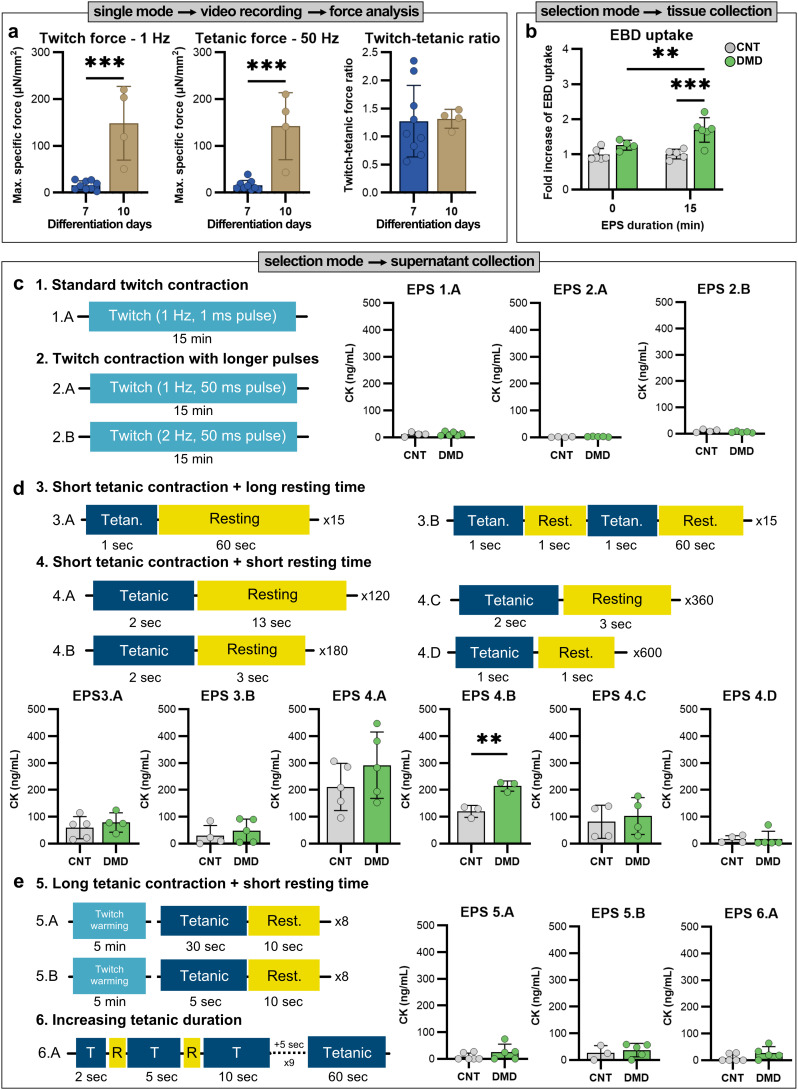
Functional validation of the Myo-MOVES using 3D skeletal muscle tissues. (a) Force evaluation in response to 1 and 50 Hz of electrical pulse stimulation (EPS) to assess the contractile performance of Duchenne muscular dystrophy (DMD) 3D tissues after 7 and 10 days of differentiation. (b) Evans blue dye (EBD) uptake (expressed as the fold increase of the mean gray value of EBD compared with non-stimulated control (CNT) levels) of CNT and DMD tissues at day 10 of differentiation after 15 min of 1 Hz EPS. (c–e) Creatine kinase (CK) release following different electrical pulse stimulation (EPS) protocols in 3D skeletal muscle tissues from control (CNT) and Duchenne muscular dystrophy (DMD) samples. Each panel includes a schematic representation of the EPS protocols and corresponding CK quantification in the supernatant after EPS by ELISA. *N* = 3–5 tissues per group. Significance was determined using the Student *t*- test or ANOVA to compare differences between the groups. Statistical test outcomes are reported as mean ± SD (**p < 0.01).

Following the differentiation period, we placed the engineered muscle tissues in the stimulation platform and applied a range of EPS regimens. The tissues were functional and capable of contracting in response to electrical stimulation (SI Video S1 and S2). As expected, low-frequency EPS induced twitch contractions (Fig. 4f), while high-frequency EPS led to tetanic-like contraction dynamics (Fig. 4g). To validate the Myo-MOVES functionality, we conducted a series of tests demonstrating the platform's versatility and flexible adaptation to diverse research needs. These representative assays address common challenges encountered in 3D muscle research laboratories. First, we explored the use of the single stimulation mode to assess contractile performance at different stages of DMD tissue differentiation (Fig. 5a). Next, we employed the selection mode to evaluate the persistence of Evans blue dye (EBD) uptake in DMD tissues following EPS at extended differentiation stages (Fig. 5b), and screen EPS protocols that induce CK release in DMD tissues (Fig. 5c–e).

As already described, Myo-MOVES can selectively stimulate individual wells without affecting neighbouring samples, enabling the recording of single samples without fatiguing the rest. This eliminates the need for users to place and stimulate tissues one by one, allowing for more efficient data acquisition. We took advantage of this characteristic to compare the maximal contractile forces generated by DMD tissues after 7 and 10 days of differentiation (Fig. 5b). Using single-pulse stimulation at 1 Hz, twitch contractions were significantly stronger in tissues differentiated for 10 days. Similarly, tetanic contractions induced by 50 Hz stimulation showed increased force after 10 days of differentiation. Despite these changes, the twitch-to-tetanic force ratio remained consistent across both time points. The Myo-MOVES platform significantly reduces experimental duration, achieving a 2.5- to 3-fold decrease in total time compared to conventional methodologies. Preparation of 14 tissue constructs on the Myo-MOVES plate requires only 5–10 minutes, and complete functional testing using six stimulation frequencies (10 seconds per stimulation with a 10-second rest period) is accomplished in approximately 28 minutes. In contrast, traditional approaches necessitate sequential handling and individual transfers of samples from the incubator, extending the total experiment time to over 80 minutes. This streamlined workflow not only enhances efficiency but also minimizes manual intervention and reduces exposure to environmental fluctuations, improving experimental consistency and reproducibility.

Myo-MOVES also provides the flexibility to stimulate multiple wells simultaneously, which is particularly advantageous for experiments focused on parameters other than contractile force. We leveraged this capability to evaluate sarcolemmal damage in DMD tissues. DMD myofibers are particularly prone to sarcolemmal rupture upon contraction, which leads to the leakage of intracellular enzymes such as creatine kinase (CK). Our preliminary studies had shown that 15 minutes of twitch contractions at 1 Hz caused damage in DMD but not CNT tissues.^22^ Sarcolemmal damage in that case was assessed using Evans blue dye (EBD), a fluorescent small molecule that penetrates cells when the membrane is disrupted. These initial experiments were conducted using tissues differentiated for 7 days. To evaluate whether sarcolemmal damage persists at later stages of maturation, we repeated the protocol in tissues differentiated for 10 days. The results mirrored those at day 7, with DMD tissues showing substantial EBD accumulation after 15 minutes of twitch stimulation (Fig. 5b), evincing the continued presence of membrane instability at later differentiation stages. This type of comparison is particularly relevant for applications such as drug testing, where mature tissues may better reflect physiological responses and disease phenotypes. In a conventional setup limited to 6-well stimulation, applying a 15-minute protocol to 24 samples required four consecutive runs, amounting to 60 minutes in total. In contrast, Myo-MOVES performs the same protocol across all 24 wells in parallel, completing the experiment in just 15 minutes and reducing variability introduced by sequential processing.

Having confirmed sarcolemmal damage *via* EBD uptake,^22^ we next used Myo-MOVES to investigate whether this disruption was accompanied by a release of CK. For this, we applied 15 minutes of twitch contractions at 1 Hz, and, one hour after stimulation, we collected the supernatant to quantify levels of CK (SI Fig. S7). However, we observed low concentrations of the protein, around 10 ng mL^−1^, in the supernatant of both CNT and DMD samples after this EPS protocol (EPS 1.A, Fig. 5c). This discrepancy with our previous results may be explained by the formation of small tears in the sarcolemma—large enough to allow EBD entry but insufficient to permit the release of larger enzymes like CK. To further explore the utility of Myo-MOVES, we investigated its capability to screen a variety of EPS protocols to identify stimulation conditions that may induce CK release in DMD tissues more effectively. We tested whether increasing the stimulation pulse duration (from 1 ms to 50 ms) or the frequency (from 1 Hz to 2 Hz) would result in CK release. Neither adjustment led to an increase of CK levels (EPS 2, Fig. 5c), suggesting that even an intensified twitch regimen was not sufficient to cause substantial sarcolemmal rupture.

We further explored the influence of high frequency EPS on the induction of sarcolemmal damage. There is evidence in the literature that tetanic contractions followed by muscle relaxation can cause fiber damage in mouse models of DMD.^53^ In dystrophin-deficient (mdx) mice, muscle fiber rupture is typically observed during quiescent periods following intense contractile stress. In those studies, 1-second tetanic contractions (125 Hz) followed by 60 seconds of rest resulted in muscle damage. We applied one or two short (1 second) high-frequency (150 Hz) tetanic contractions followed by long resting periods (60 seconds) (EPS 3, Fig. 5d). These regimens led to a slight increase in CK release compared to the twitch-only protocols, although this increase was observed in both CNT (59.23 and 29.13 ng mL^−1^) and DMD tissues (78.78 and 48.21 ng mL^−1^).

Next, we tested more repetitive regimens consisting of short tetanic contractions (1 or 2 seconds) followed by shorter resting intervals (1, 3, or 13 seconds), repeated for 15, 20, or 30 minutes. The most prominent CK release was observed after applying 2-second tetanic contractions followed by 13 seconds of rest for 30 minutes (120 repetitions), which resulted in CK levels exceeding 200 ng mL^−1^ in the supernatant (EPS 4.A, Fig. 5d). Although DMD samples showed a trend toward higher CK release (291.77 *vs.* 211.01 ng mL^−1^), the difference was not statistically significant in this case. Interestingly, we observed a significant increase in CK levels specifically in DMD tissues (214.76 ng mL^−1^) compared to CNT (120.07 ng mL^−1^) when the rest period was reduced to 3 seconds and the number of repetitions increased to 180 over 15 minutes (EPS 4.B, Fig. 5d). This indicates that DMD tissues showed greater susceptibility to sarcolemmal membrane damage. However, further intensification of the EPS regimen led to a decrease in CK levels. For example, we detected 81.29 ng mL^−1^ in CNT and 102.47 ng mL^−1^ in DMD samples when applying the same regimen for 30 minutes (EPS 4.C, Fig. 5d). Decreasing both contraction and relaxation times to 1 second but increasing the repetitions to 600 (20 minutes) (EPS 4.D, Fig. 5d) resulted in even reduced CK release, obtaining levels near the limit of detection. We hypothesise that the observed reduction in CK levels under more intense EPS regimens may result from increased electrolysis at the electrodes, which could alter the local microenvironment, such as increasing temperature or shifting pH, ultimately affecting CK stability or its measurable release into the supernatant.

We also evaluated the effect of longer tetanic contractions (5 and 30 seconds), each followed by 10 seconds of rest, after an initial 5-minute twitch warm-up (3 minutes at 1 Hz followed by 2 minutes at 2 Hz) (EPS 5, Fig. 5e). These conditions did not cause a significant increase in CK levels in either CNT (8.53 and 26.7 ng mL^−1^) or DMD samples (25.20 and 36.8 ng mL^−1^). Finally, we explored a progressive EPS protocol in which tetanic contraction duration increased incrementally from 2 seconds up to 60 seconds (80 Hz), with each contraction followed by 10 seconds of rest (EPS 6.A, Fig. 5e). Again, we did not detect significant increases in CK release (8.94 ng mL^−1^ for CNT supernatants and 27.08 ng mL^−1^ for DMD). These results suggest that long, continuous tetanic contractions are not effective at inducing sarcolemmal rupture in this system.

The Myo-MOVES platform enabled us to systematically explore a wide range of EPS parameters to identify those that could induce the sarcolemmal damage associated with DMD. The platform allowed for straightforward and flexible configuration, as we could place the tissues directly onto the device and easily select specific wells for stimulation. Among the protocols tested, short tetanic contractions followed by resting led to measurable CK release in 3D skeletal muscle tissues. Among the conditions tested, a 15-minute protocol consisting of 2-second tetanic contractions with 3 seconds of rest resulted in a significant difference in CK release between CNT and DMD samples. To the best of our knowledge, this is the first time that increased CK levels have been detected in DMD 3D skeletal muscle tissues after contractile stress. While the small sample size in a single experimental series limits definitive conclusions, this exploratory work suggests that contraction-induced stress can elicit sarcolemmal damage in DMD 3D tissues. These findings are preliminary and require confirmation in future independent experiments. However, the use of the Myo-moves platform proved highly valuable for efficiently screening the effects of multiple EPS regimens within the same experimental setup, enabling rapid identification of stimulation conditions that elicit distinct physiological responses. This was made possible by the platform's capacity to fine-tune EPS parameters and by validating damage using a sensitive ELISA-based assay, in contrast to previous studies that relied on enzymatic activity assays and non-optimised EPS.^43^ Moreover, while others have measured CK release using chemical injury models,^54^ our approach provides a physiologically relevant alternative based on functional contraction-induced stress. These proof-of-concept results highlight the potential of this platform to model disease-relevant phenotypes.

## Conclusions

Here we present Myo-MOVES, an electric stimulation platform designed for 3D skeletal muscle tissues. The Myo-MOVES platform allows for the controlled electric pulse stimulation of up to 24 tissues simultaneously, with the possibility of applying different stimulation regimens. Our system presents several virtues, including versatility, high throughput, ease of use and low cost. The first part, the selector, allows the user to choose a single or a selection of samples to stimulate. An informative dashboard makes the device exceptionally intuitive. The second part, the stimulator, delivers the stimulation signal to the tissues in a commercial 24-well culture plate. The separation between electrodes maximises the available space in the well for the use of samples of different sizes, and the holes in the PCB and its support allow imaging during experimentation.

Future improvements for this system could include the use of biphasic stimulation signals to reduce hydrolysis, decreasing the potential damage to the samples or analytes. Furthermore, the signal generation and branching could be integrated, achieving a miniaturised device with increased portability. This stimulation system could also be used on other commercial culture plates by slightly adapting the design of the stimulator PCB holder. The stimulator could also be adapted for microfluidic systems. By integrating and interconnecting the electrodes in a muscle-on-a-chip, experiments other than endpoint measurements could be made using this system.

We utilised Myo-MOVES for the *in vitro* analysis of a 3D skeletal muscle model of DMD and demonstrated its broader utility for phenotypic characterisation of engineered muscle tissues. First, the platform allowed us to detect maturation-dependent increases in twitch and tetanic responses. Second, Myo-MOVES facilitated the evaluation of membrane integrity through EBD uptake, confirming sarcolemmal fragility as a hallmark of DMD tissues. Finally, the platform was key in exploring electrical stimulation protocols for inducing CK release. Our findings suggest that DMD 3D skeletal muscle tissues can reproduce the characteristic membrane fragility of the disease. While twitch contractions were unable to significantly trigger CK release, inducing the full contraction of tissues through tetanic contractions and allowing their complete relaxation with sufficient resting time led to a marked increase. This points to contraction-induced movement as a key contributor to muscle damage. The Myo-MOVES platform played an essential role in the screening of this phenotype, as it enabled straightforward testing and refinement of EPS protocols. These experiments represent preliminary data aimed at demonstrating the ability of the Myo-MOVES platform to capture physiologically relevant phenotypes. While the observed differences between systems fall within expected biological variability, a comprehensive assessment of reproducibility and inter-sample variability of CK release in DMD *in vitro* 3D tissues is beyond the scope of this study and will be addressed in future work. These results highlight the potential of Myo-MOVES as a versatile tool for studying not only neuromuscular disorders, but also contractility-associated pathologies in other tissues, such as cardiac muscle.

## Author contributions

Martín Ruiz-Gutiérrez: conceptualization, investigation, methodology, formal analysis, visualisation, writing – original draft. Ainoa Tejedera-Villafranca: conceptualization, investigation, methodology, formal analysis, visualisation, writing – original draft. Sergi Pujol-Pinto: investigation and methodology. Javier Ramón-Azcón: conceptualization, supervision, writing – review & editing, funding acquisition. Juan M. Fernández-Costa: conceptualization, supervision, visualization, writing – original draft, writing – review & editing, funding acquisition.

## Conflicts of interest

There are no conflicts to declare.

## Supplementary Material

LC-025-D5LC00614G-s001

LC-025-D5LC00614G-s002

LC-025-D5LC00614G-s003

LC-025-D5LC00614G-s004

## Data Availability

Supplementary information is available: The PDF contains CK ELISA optimization protocol and buffers, measured stimulation signals at different frequencies in the selector, the stimulator, a custom conventional stimulation plate and a commercial stimulation plate, a comparison of muscle contraction behaviour between the Myo-MOVES and a conventional plate, representative confocal images of a human 3D skeletal muscle tissue, optimization data of the CK ELISA, and the list of schematics of the developed electronics and 3D parts, including their designation and description, as well as the schematics themselves. Supplementary videos 1 and 2: Twitch and tetanic muscle contraction under low and high frequency stimulation, respectively, using the Myo-MOVES platform. Microscopy recordings are then processed to calculate contraction force based on pillar deflection. Table 1: Total cost of the developed parts of the Myo-Moves platform, broken down by component. See DOI: https://doi.org/10.1039/D5LC00614G. The data supporting this article have been included as part of the SI.
