# Supplementary material for: Myo-MOVES: a custom electrical stimulation system for functional studies of 3D bioengineered muscle
Source: Lab Chip. 2025 Oct 1;25(21):5677–90. doi: 10.1039/d5lc00614g (PMC12487923; doi:10.1039/d5lc00614g)
Supplement: LC-025-D5LC00614G-s004 [file LC-025-D5LC00614G-s004.pdf]

Supplementary Information (SI) for Lab on a Chip.
This journal is © The Royal Society of Chemistry 2025

Supplementary information

Myo-MOVES: a custom electrical stimulation system for functional studies of 3D bioengineered muscle

Martín Ruiz-Gutierrez*, Ainoa Tejedera-Villafranca*, Sergi Pujol-Pinto ,
Javier Ramón-Azcón and Juan M. Fernández-Costa

<u>Supplementary methods</u>

Optimization of Enzyme-Linked ImmunoSorbent Assay (ELISA) for the detection of Creatin Kinase

To detect creatine kinase in the supernatant of the 3D skeletal muscle tissues, a sandwich ELISA assay was developed. First, antibody concentrations were optimized by testing the capture antibody (Recombinant Anti-Creatine Kinase MM antibody, Abcam) at various concentrations. The capture antibody was diluted in coating buffer (Na_2CO_3 15 mM, $NaHCO_3$ 35 mM, pH 9,6 adjusted with HCl and NaOH) and incubated overnight at 4°C. Plates were washed using a Microplate Washer (BioTek 405 TS) with PBST (PBS 1x + Tween 1%). Following a 2-hour blocking step with a blocking solution (1% BSA-PBS 1x), Recombinant Human Creatine Kinase MM/CKMM Protein was added at 1 µg/mL and incubated for 1 hour at room temperature. Subsequently, the secondary antibody (Human Creatine Kinase M-type (CKMM) Antibody Biotin Conjugate) was added at various concentrations for 1 hour at room temperature. Two different streptavidin–peroxidase solutions were tested (Streptavidin–Peroxidase from Streptomyces avidinii, Sigma-Aldrich and Streptavidin poly-HRP (horseradish peroxidase) Pierce™, Thermo Fisher) at 0.1 µg/mL for 30 minutes at room temperature. Following washes, the substrate solution (11,76 mL of citrate buffer (10 mM sodium citrate tribasic dihydrate, pH 4 adjusted with acetic acid), 192 µL of TMB (6 mg/mL in DMSO) and 48 µL of H_2O_2 1%) was incubated for 30 minutes covered from the light. The reaction was stopped by adding 50 µL of 2N H_2SO_4, and absorbance was measured in a spectrophotometer at 450 nm.

The chosen concentrations after the optimization process were 0,625 µg/mL of capture antibody, 1,25 µg/mL of biotinylated antibody and 0,1 µg/mL of streptavidin conjugated to poly-HRP, which was selected due to its superior performance (Supp. Fig 6).

Supplementary figures

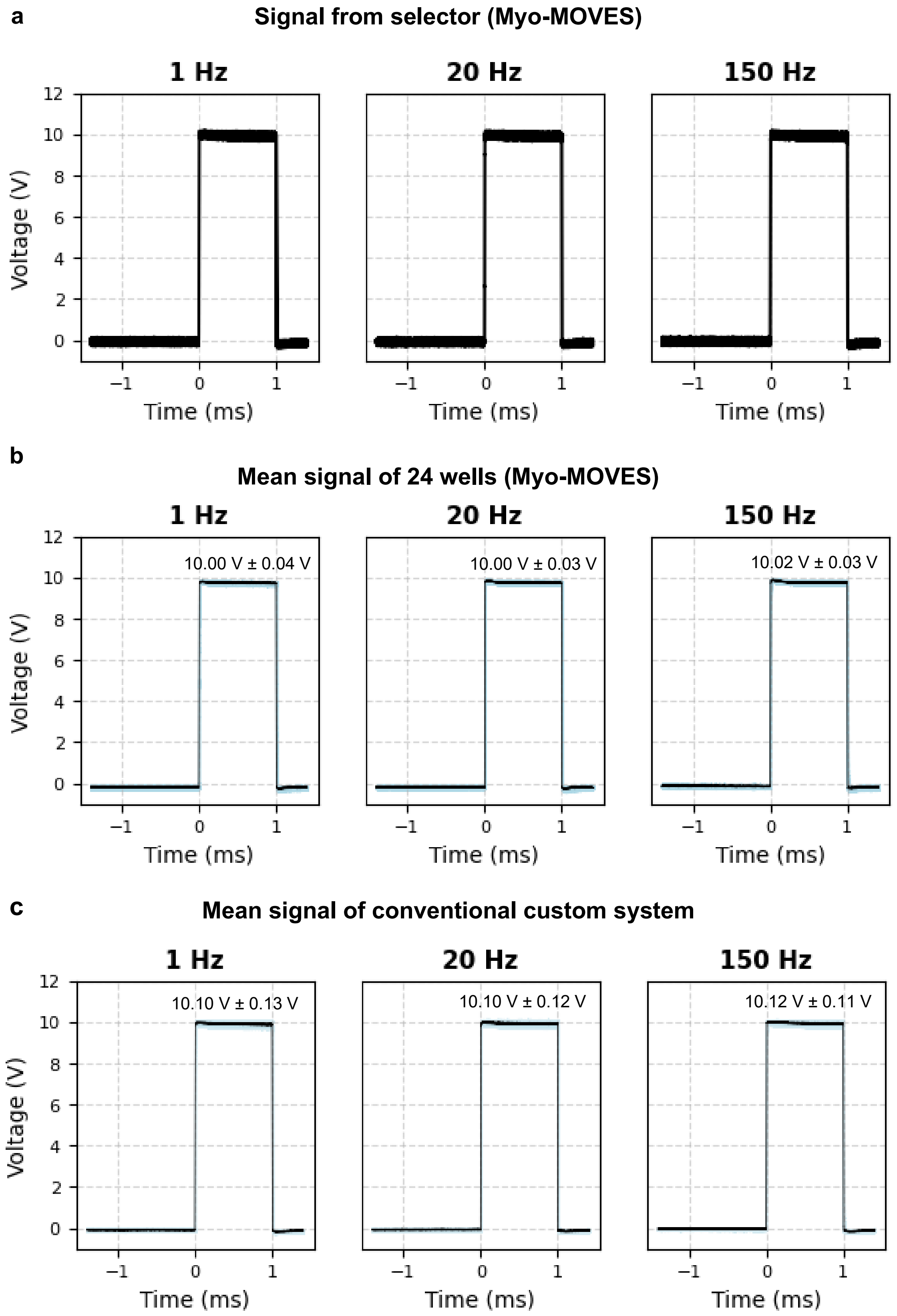

d

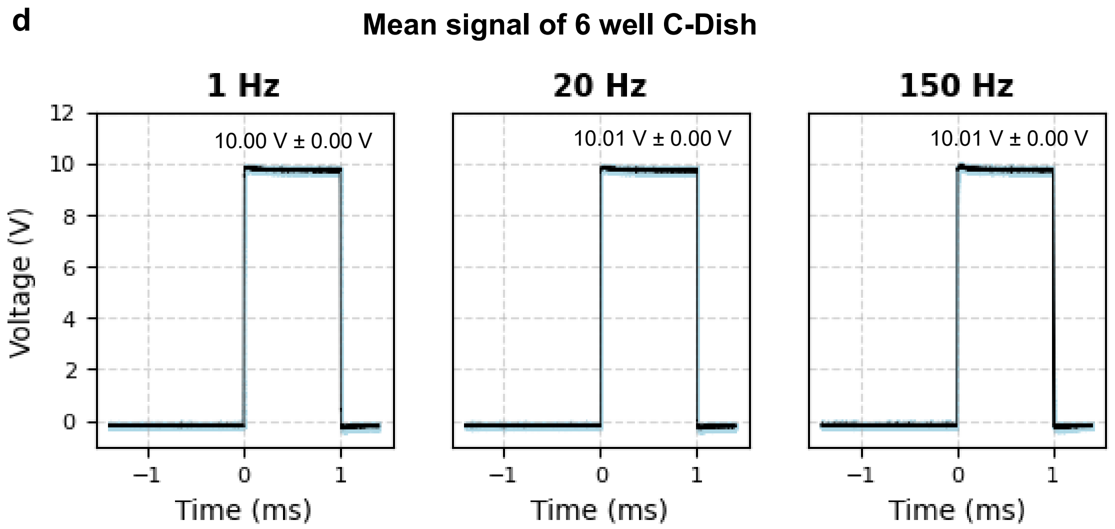

Supp. Fig. 1. Voltage traces recorded during electrical stimulation at different frequencies (1 Hz, 20 Hz, and 150 Hz) in the Myo-MOVES device (both the selector (a) and in the 24-well plate (b) (n=24), a conventional custom system (c) and commercially available C-Dish (d) (n = 6). In b-d, each panel shows the average voltage signal (black line) measured across all multiple wells for a given stimulation frequency. Signals were recorded using an oscilloscope and smoothed using a Savitzky–Golay filter. Shaded regions represent the standard deviation (±SD) across all recordings.

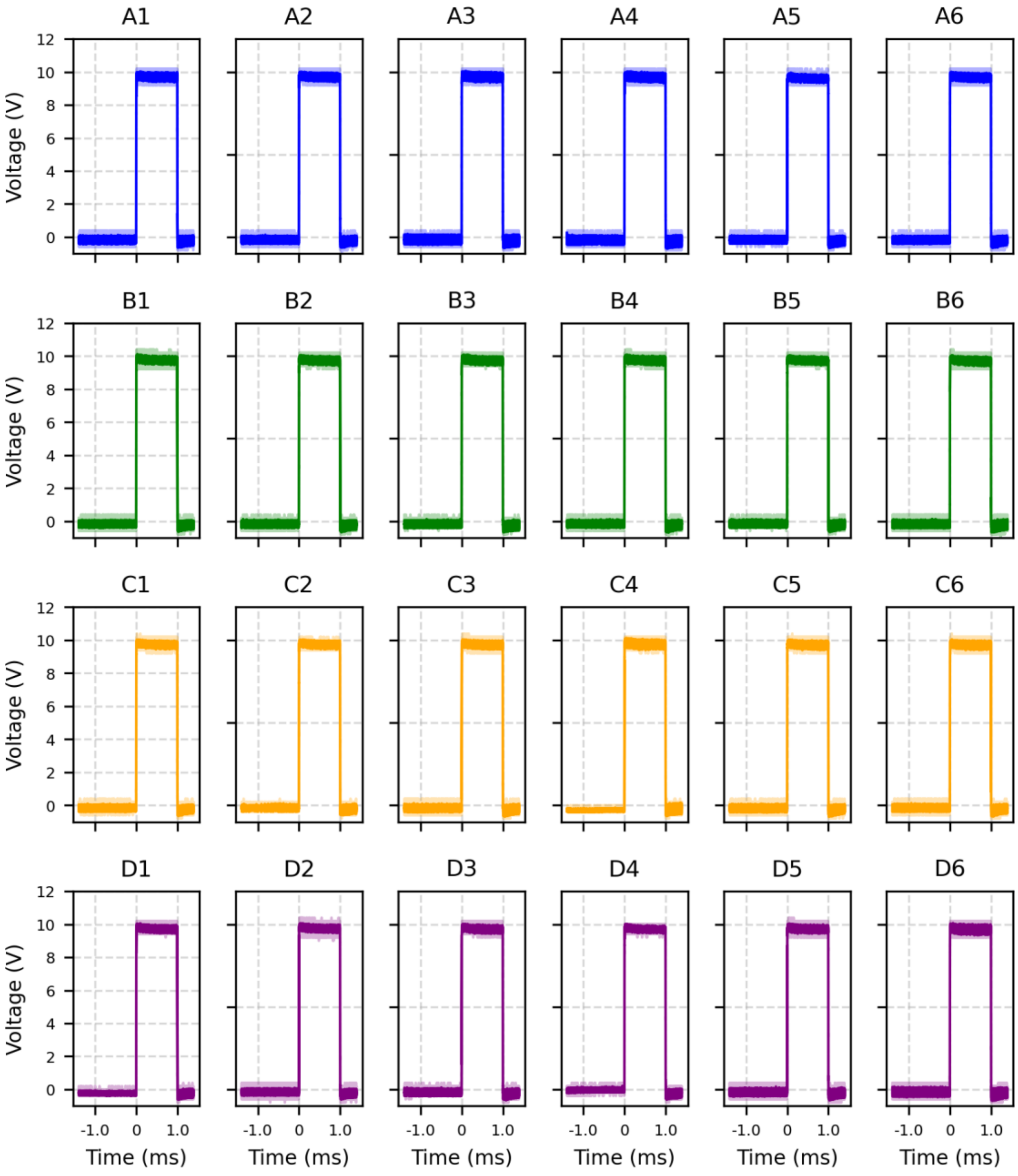

Supp. Fig. 2. Voltage traces recorded during electrical stimulation at 1 Hz in every well of the stimulation plate of the Myo-MOVES device. Signals were recorded using an oscilloscope and smoothed using a Savitzky–Golay filter.

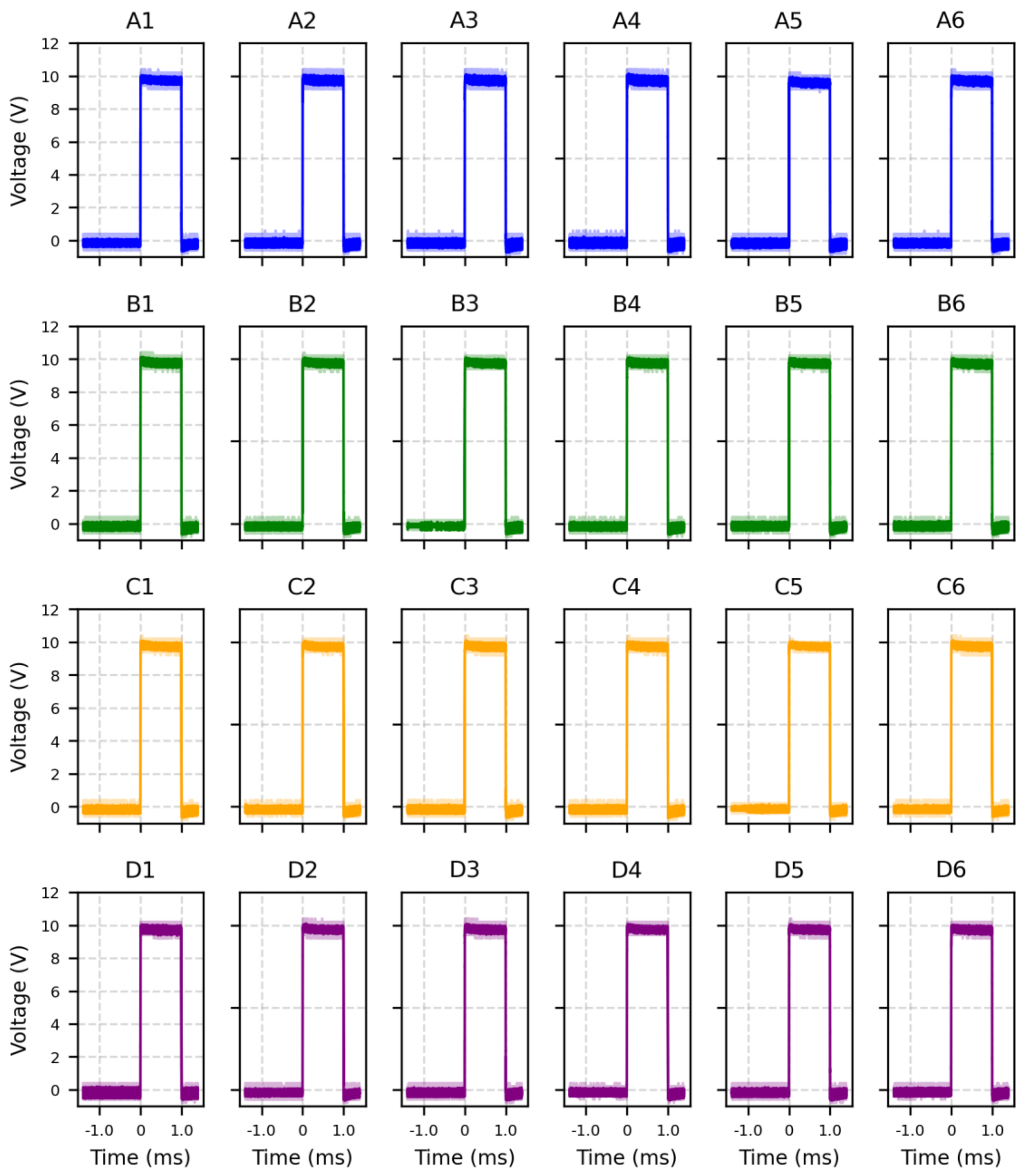

Supp. Fig. 3. Voltage traces recorded during electrical stimulation at 20 Hz in every well of the stimulation plate of the Myo-MOVES device. Signals were recorded using an oscilloscope and smoothed using a Savitzky–Golay filter.

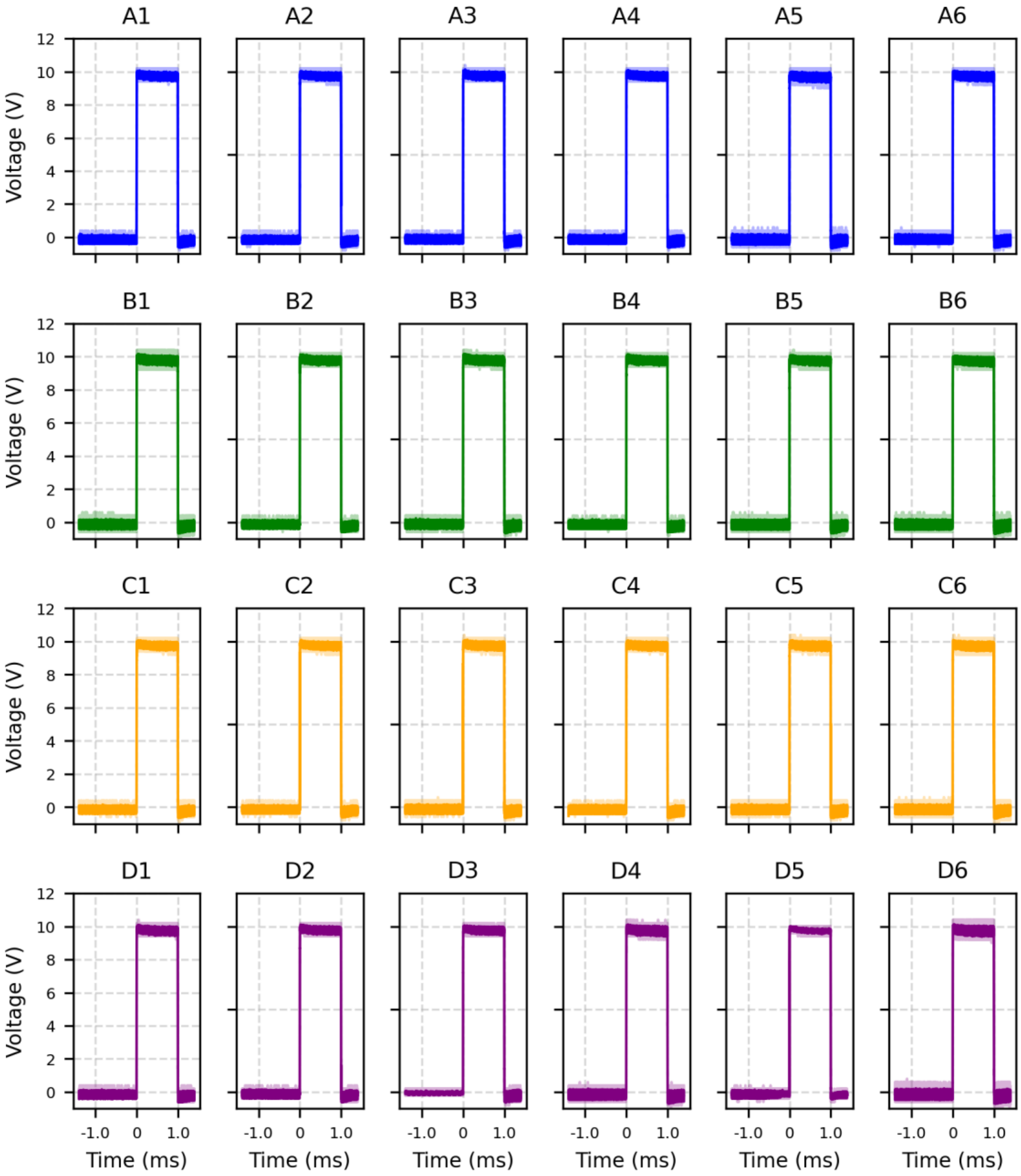

Supp. Fig. 4. Voltage traces recorded during electrical stimulation at 150 Hz in every well of the stimulation plate of the Myo-MOVES device. Signals were recorded using an oscilloscope and smoothed using a Savitzky–Golay filter.

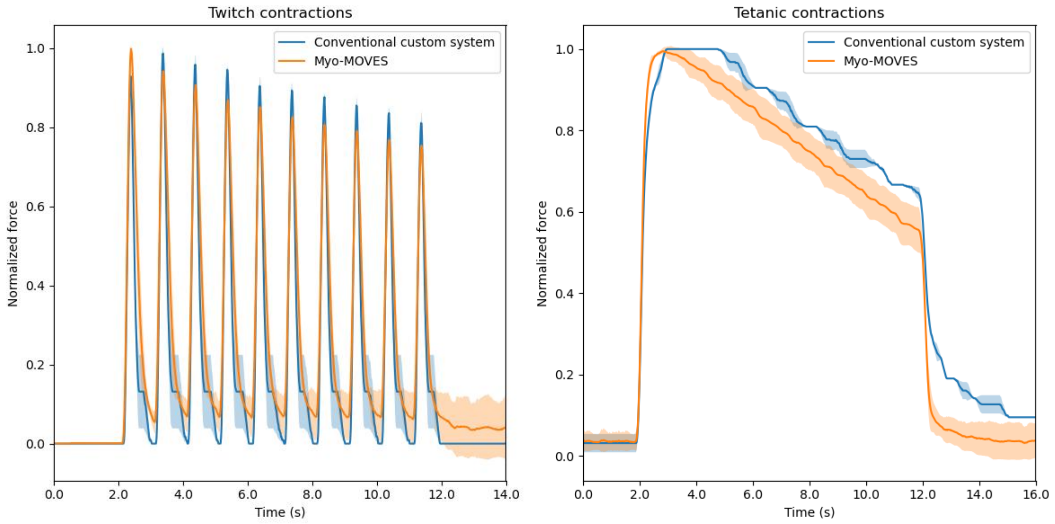

Supp. Fig. 5. Comparison of contraction profiles between Myo-MOVES and a conventional custom system. Mean normalized force traces (± standard deviation) from healthy engineered skeletal muscle tissues in response to identical stimulation protocols at 1 Hz and 25 Hz. N=3-5.

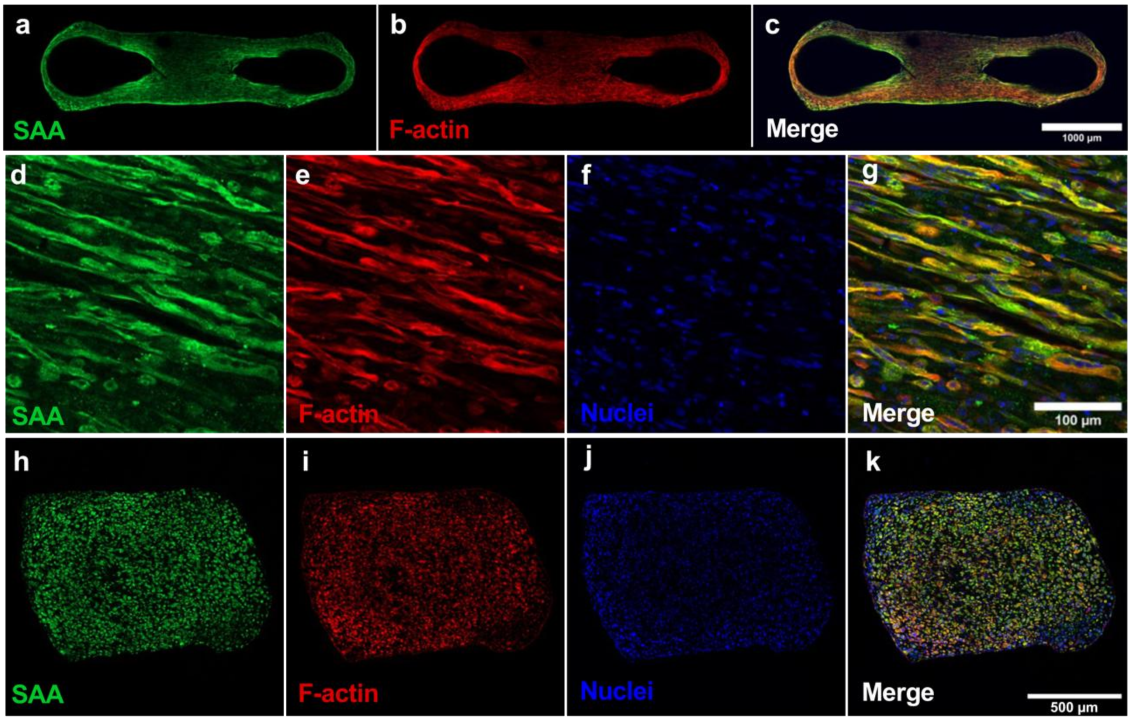

Supp. Fig. 6. Representative confocal images of a human 3D skeletal muscle tissue after seven days of differentiation, stained for sarcomeric α-actinin (SAA, green), F-actin (red), and nuclei (blue). (a–c) Whole-mount views of the tissue. Scale bar = 1000 µm. (d–g) Higher magnification views of selected regions. Scale bar = 100 µm. (h–k) Transverse cross-sections of the tissue. Scale bar = 500 µm.

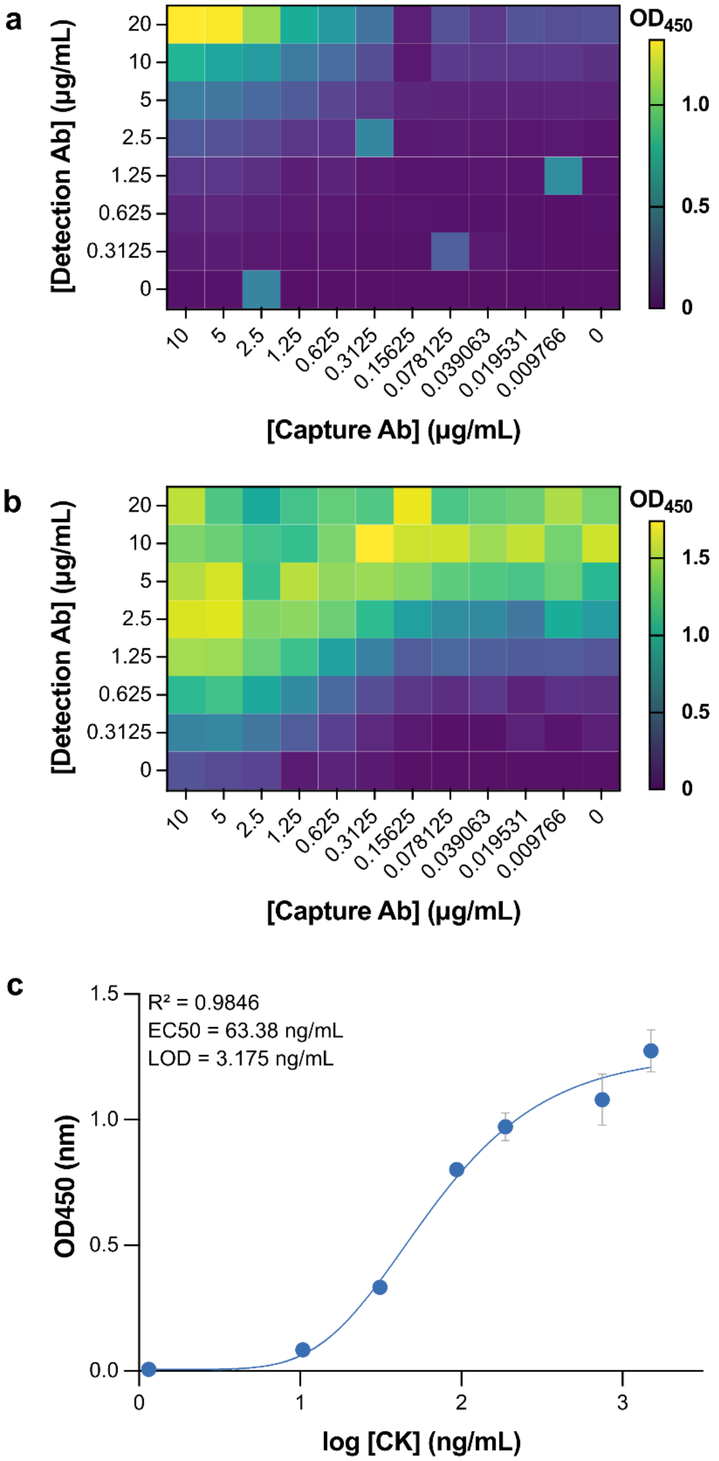

Supp. Fig. 7. Optimization of the Enzyme-Linked Immunosorbent Assay (ELISA) for the detection of creatine kinase. (a–b) Heat maps showing absorbance values for capture and detection antibody concentration combinations using HRP (a) or poly-HRP (b) conjugates. (c) Calibration curve of the optimized ELISA, indicating the coefficient of determination (R^2), half-maximal effective concentration (EC_{50}), and limit of detection (LOD).

Supplementary videos

Supp. Video 1. Representative brightfield video of twitch contraction of a 3D skeletal muscle tissue responding to low electrical pulse stimulation (1 Hz).

Supp. Video 2. Representative brightfield video of tetanic contraction of a 3D skeletal muscle tissue responding to high electrical pulse stimulation (50 Hz).

Supplementary table

Supp. Table 1. Detailed costs of the Myo-MOVES platform components.

Supplementary schematics

Electronic schematics list

Designation	Description
**A0.1**	Selector
**A0.2**	Selector PCB
**A1.1**	Stimulator
**A1.2**	Stimulator PCB

Footnotes:

- A0.1: Electronic schematic of the selector
- A0.2: Blueprint of the selector PCB
- A1.1: Electronic schematic of the stimulator
- A1.2: Blueprint of the stimulator PCB

Plans list

Selector:

Designation	Description
**B0**	Selector box base
**B1**	Selector box lid
**B2**	Selector box rear lid
**B3**	Displays window
**B4**	LEDs window
**B5**	M4 screw stand
**B6**	Selector assembly

Stimulator:

Designation	Description
**C0**	Electrode
**C1**	Stimulator PCB
**C2**	Stimulator support
**C3**	Stimulator support base
**C4**	Stimulator connector cover
**C5**	Oscilloscope probes connector cover
**C6**	Stimulator PCB assembly
**C7**	Stimulator assembly

Tools:

Designation	Description
**D0**	Longitudinal electrode cutting support
**D1**	Transversal electrode cutting support
**D2**	Electrode cutting lever
**D3**	Electrode mounting guide

Note: The drawings of the Thermo Fisher Nunc 24 well plate and PDMS moulds and rings are not included as they were used for illustrative purposes only.

Footnotes:

Selector

- B0: Drawing of the base of the selector box
- B1: Drawing of the lid of the selector box
- B2: Drawing of the rear lid of the selector box
- B3: Drawing of the window for the 7-segment displays of the dashboard
- B4: Drawing of the window for the LEDs of the dashboard
- B5: Drawing of the pads of the selector box
- B6: Exploded assembly view drawing of the selector box with bill of materials

Stimulator:

- C0: Drawing of the graphite electrode
- C1: Drawing of the stimulator PCB
- C2: Drawing of the top part of the support of the stimulator
- C3: Drawing of the bottom part of the support of the stimulator
- C4: Drawing of the cover of the conductive parts of the stimulator connector
- C5: Drawing of the cover of the conductive parts of the connector for the oscilloscope probes
- C6: Exploded assembly view drawing of the stimulator PCB and the 48 electrodes
- C7: Exploded assembly view drawing of the stimulator, including the samples and commercial culture plate

Tools:

- D0: Drawing of the guide used to make longitudinal cuts in the graphite plates
- D1: Drawing of the guide used to make transversal cuts in the already cut longitudinally cut pieces of graphite
- D2: Drawing of the tool used to apply lever force to cut the slotted pieces of graphite
- D3: Drawing of the guide used to align and mount the electrodes in the stimulator PCB

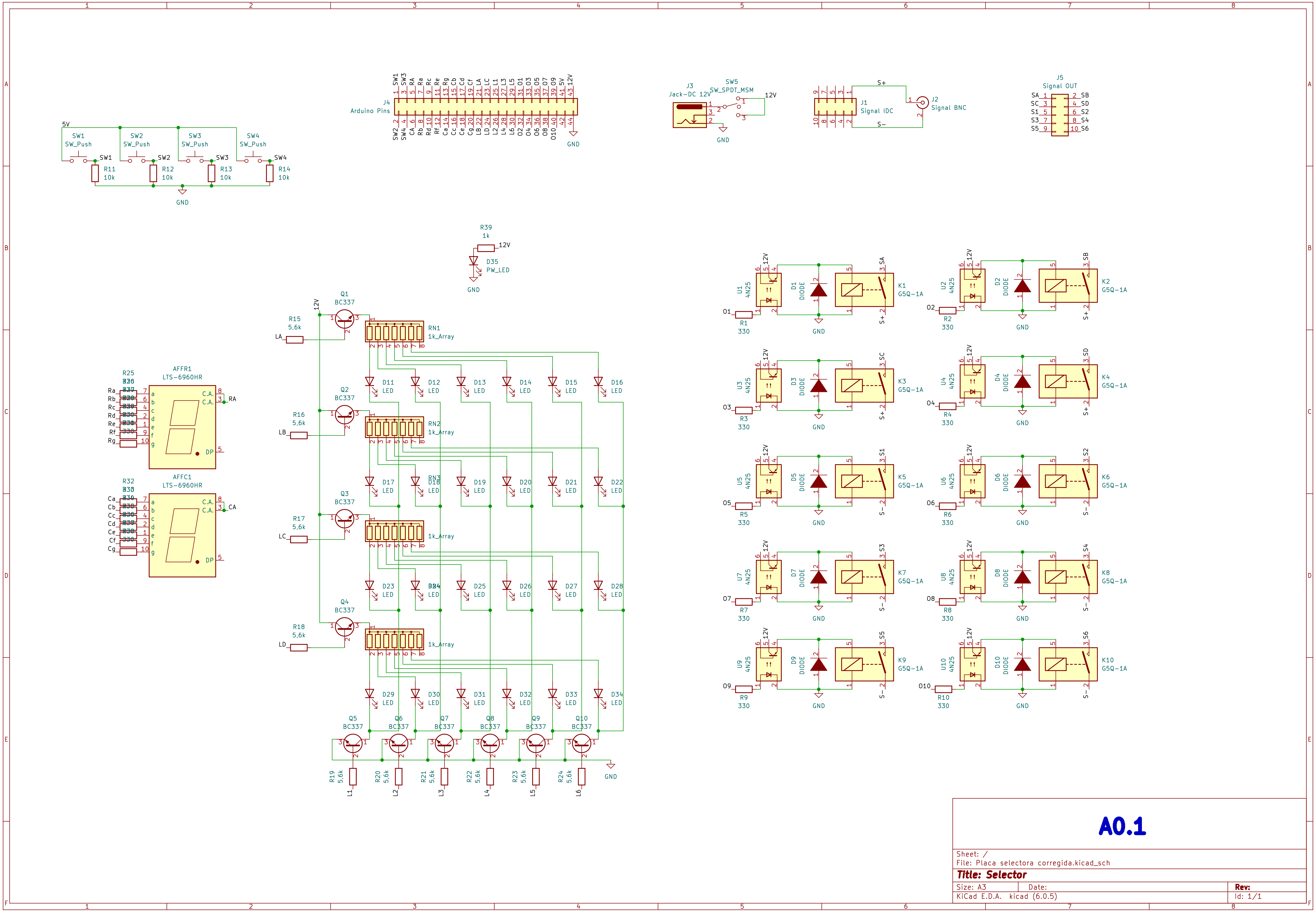

SW1 SW_Push
SW2 SW_Push
SW3 SW_Push
SW4 SW_Push
5V
R11 10k
R12 10k
R13 10k
R14 10k
GND
J4 Arduino Pins
GND
J3 Jack-DC 12V
SW5 SW_SPDT_MSM
12V
J1 Signal IDC
S+
S-
J2 Signal BNC
J5 Signal OUT
SA SB SC SD S1 S2 S3 S4 S5 S6
R39 1k
D35 PW_LED
Q1 BC337
Q2 BC337
Q3 BC337
Q4 BC337
R15 5,6k
R16 5,6k
R17 5,6k
R18 5,6k
LA LB LC LD
RN1 1k_Array
RN2 1k_Array
AFFR1 LTS-6960HR
AFFC1 LTS-6960HR
C.A.
DP
RA
CA
Q5 BC337
Q6 BC337
Q7 BC337
Q8 BC337
Q9 BC337
Q10 BC337
R19 5,6k
R20 5,6k
R21 5,6k
R22 5,6k
R23 5,6k
R24 5,6k
L1 L2 L3 L4 L5 L6
4N25
DIODE
G5Q-1A
330
A0.1
Sheet: /
File: Placa selectora corregida.kicad_sch
Title: Selector
Size: A3
Date:
Rev:
KiCad E.D.A. kicad (6.0.5)
Id: 1/1

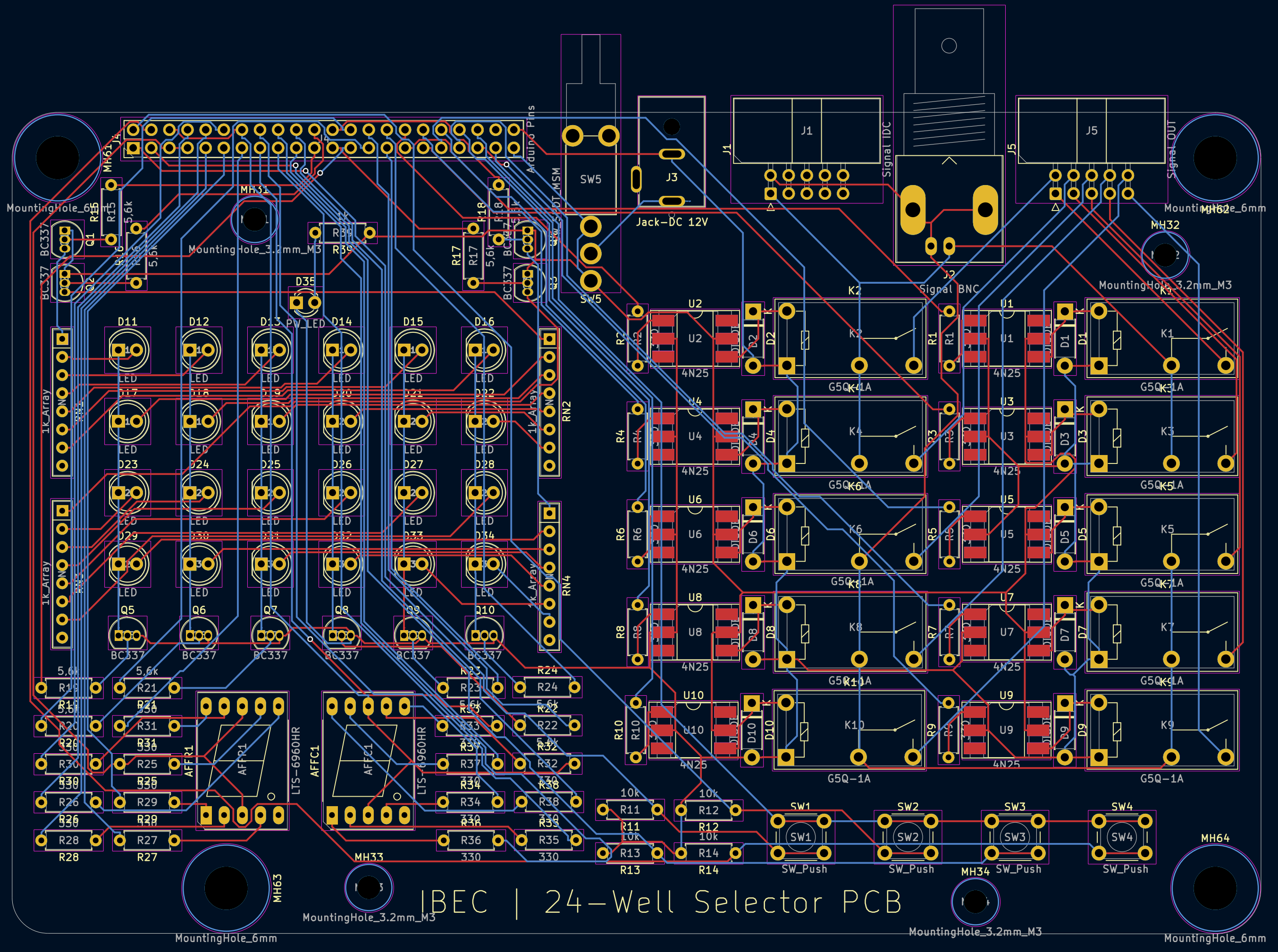

A0.2

Sheet:
File: Placa selectora corregida.kicad_pcb

Title: Selector PCB

Size: A4	Date:	**Rev:**
KiCad E.D.A. kicad (6.0.5)		Id: 1/1

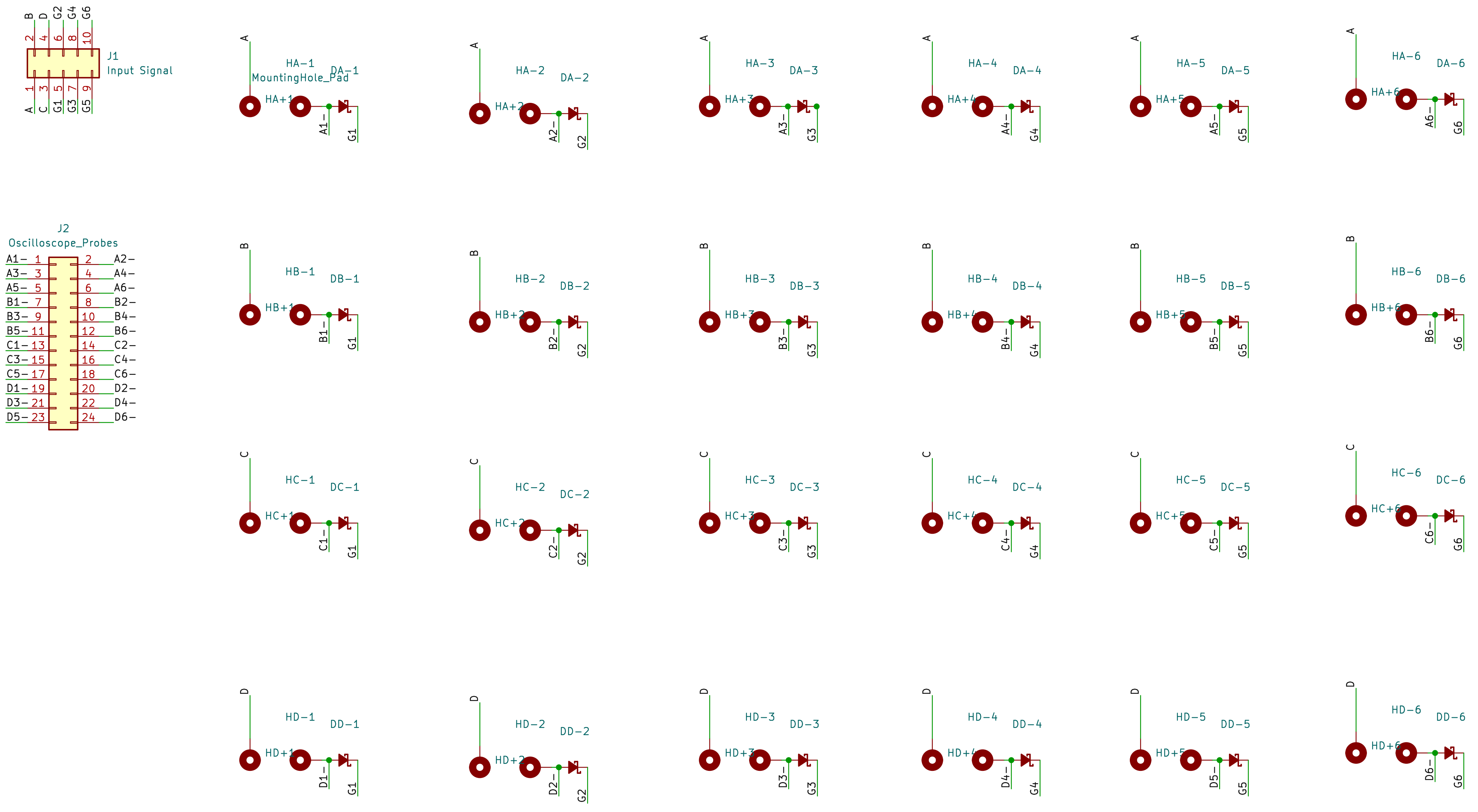

A1.1

Sheet: /
File: Placa estimuladora v2.kicad_sch

Title: Stimulator

Size: A4	Date:	Rev:
KiCad E.D.A. kicad (6.0.5)		Id: 1/1

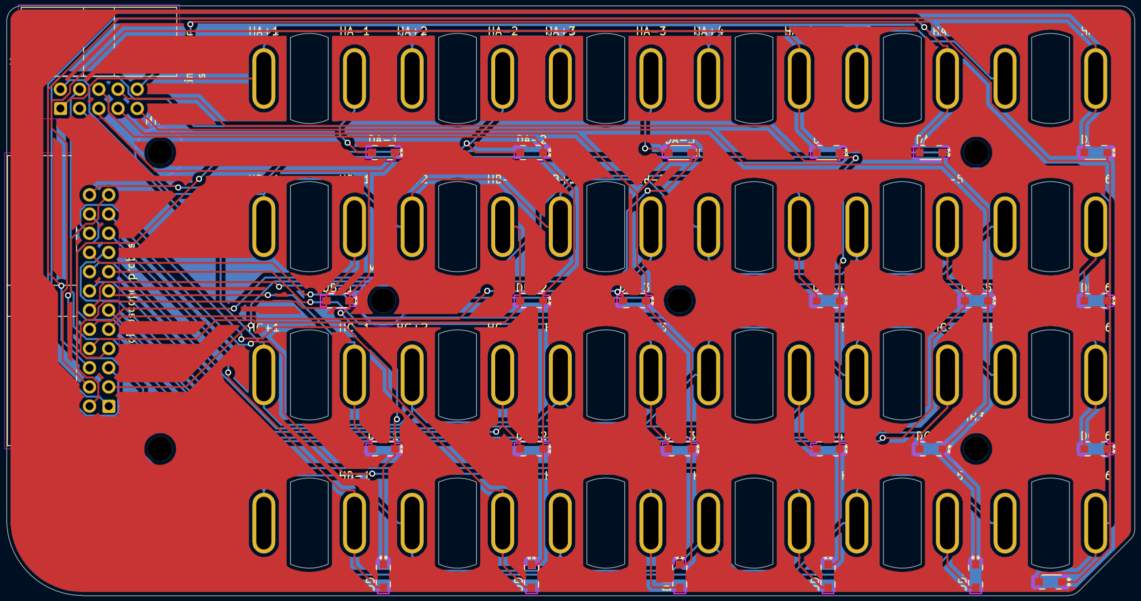

A1.2

Sheet:
File: Placa estimuladora v2.kicad_pcb

Title: Stimulator PCB

Size: A4 Date: Rev:

KiCad E.D.A. kicad (6.0.5) Id: 1/1

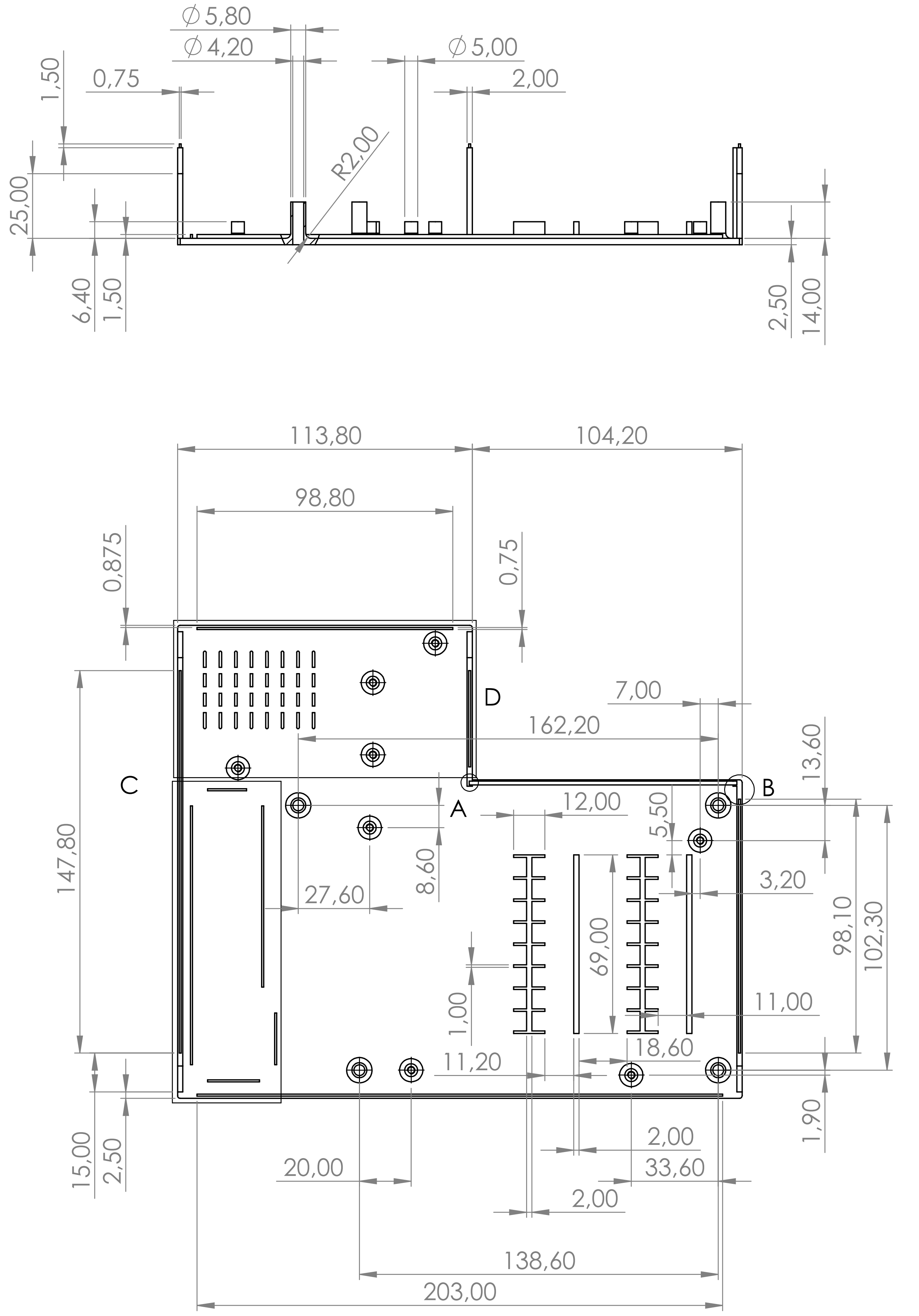

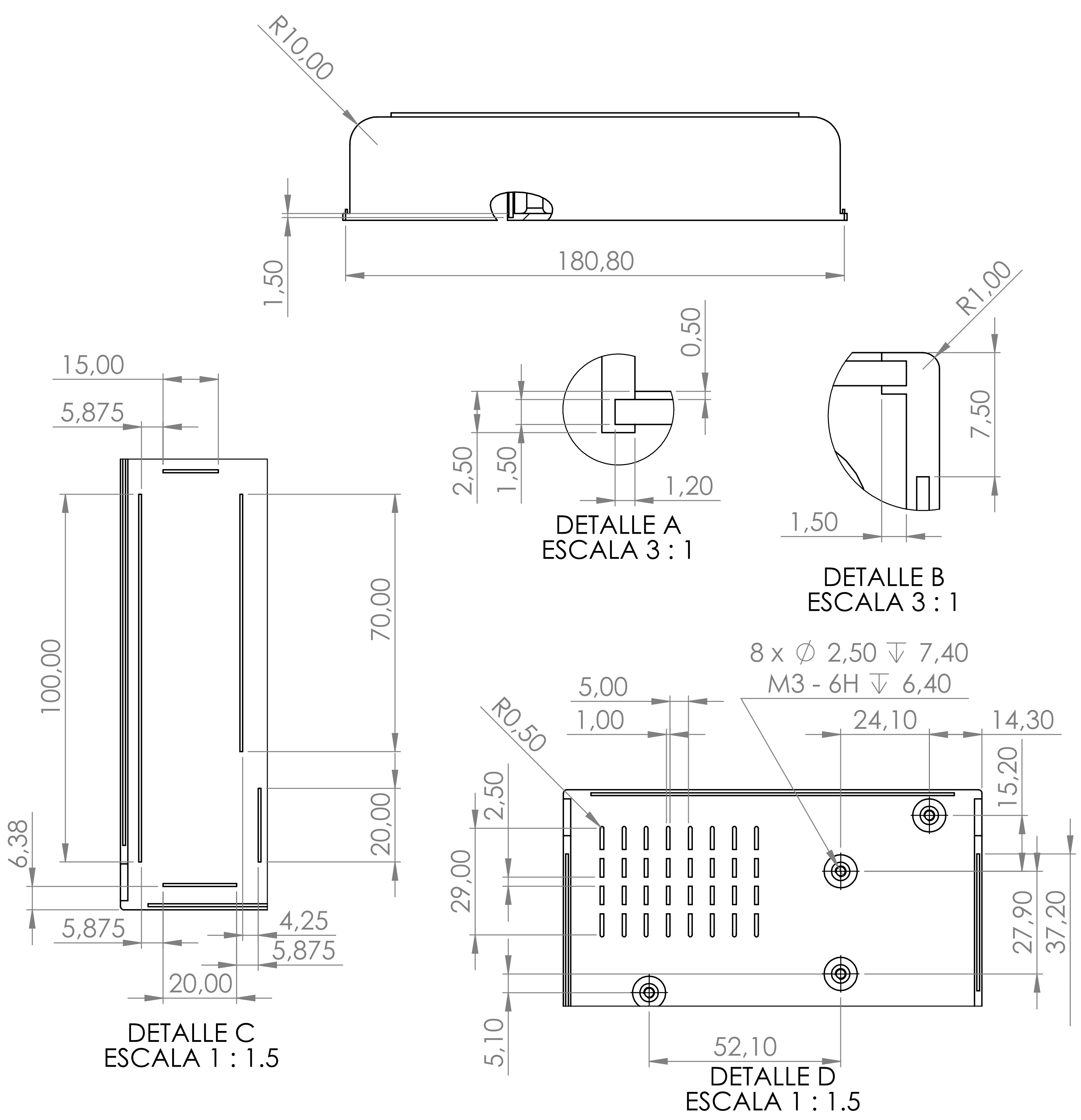

SI NO SE INDICA LO CONTRARIO: LAS COTAS SE EXPRESAN EN MM ACABADO SUPERFICIAL: TOLERANCIAS: LINEAL: ANGULAR:	ACABADO:	REBARBAR Y ROMPER ARISTAS VIVAS	NO CAMBIE LA ESCALA	REVISIÓN

	NOMBRE	FIRMA	FECHA
DIBUJ.	Martín Ruiz Gutiérrez		
VERIF.			
APROB.			
FABR.			
CALID.			

TÍTULO:

Selector box base

MATERIAL: PLA

N.º DE DIBUJO: B0

A3

PESO:

ESCALA:1:2

HOJA 1 DE 1

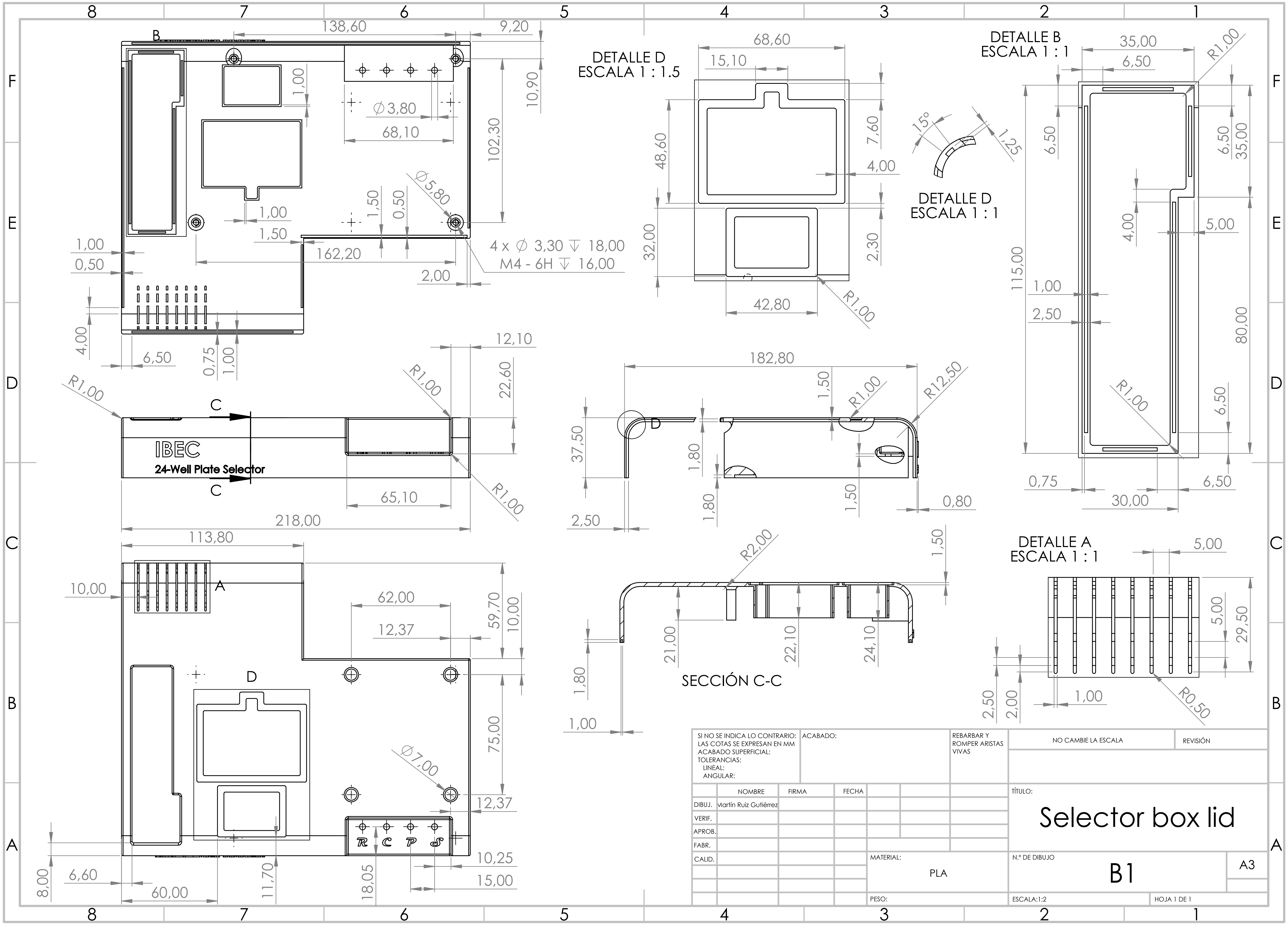

SI NO SE INDICA LO CONTRARIO: LAS COTAS SE EXPRESAN EN MM ACABADO SUPERFICIAL: TOLERANCIAS: LINEAL: ANGULAR:	ACABADO:			REBARBAR Y ROMPER ARISTAS VIVAS	NO CAMBIE LA ESCALA	REVISIÓN
	NOMBRE	FIRMA	FECHA		TÍTULO:	
DIBUJ.	Martín Ruiz Gutiérrez				Selector box lid	
VERIF.						
APROB.						
FABR.						
CALID.			MATERIAL: PLA		N.º DE DIBUJO B1	A3
			PESO:		ESCALA:1:2	HOJA 1 DE 1

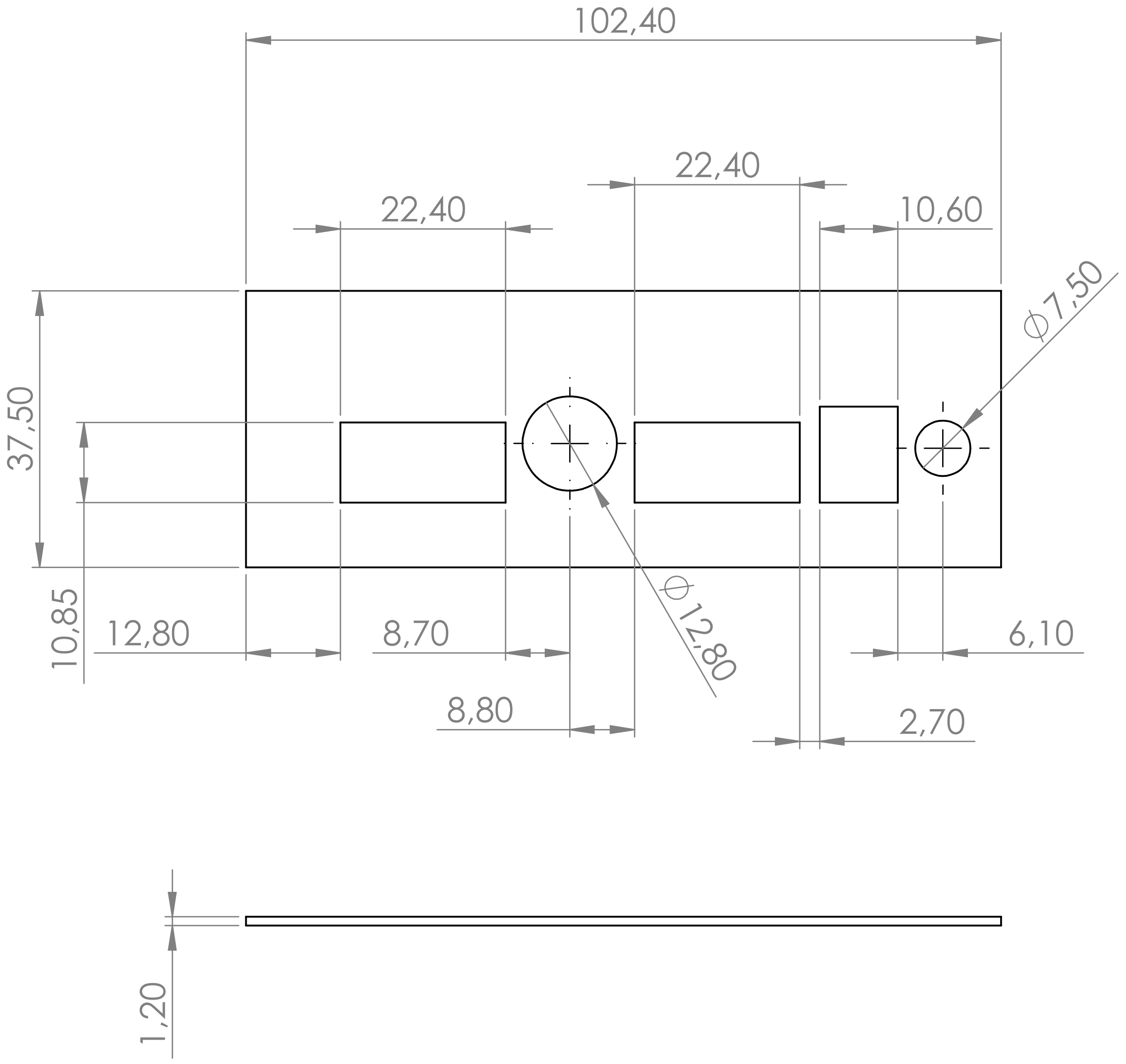

SI NO SE INDICA LO CONTRARIO:
LAS COTAS SE EXPRESAN EN MM
ACABADO SUPERFICIAL:
TOLERANCIAS:
LINEAL:
ANGULAR:

ACABADO:

REBARBAR Y ROMPER ARISTAS VIVAS

NO CAMBIE LA ESCALA

REVISIÓN

	NOMBRE	FIRMA	FECHA
DIBUJ.	Martín Ruiz Gutiérrez		
VERIF.			
APROB.			
FABR.			
CALID.			

TÍTULO:

Selector box rear lid

MATERIAL:

PLA

N.º DE DIBUJO

B2

A4

PESO:

ESCALA:1:1

HOJA 1 DE 1

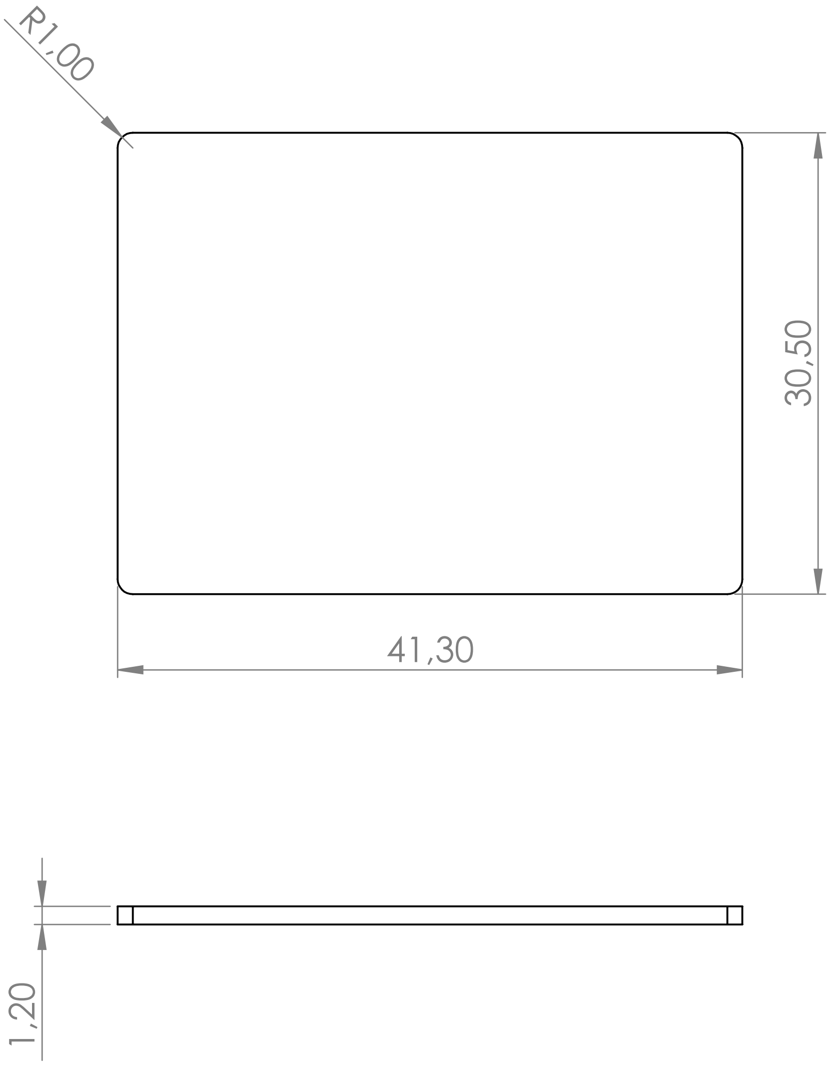

SI NO SE INDICA LO CONTRARIO:
LAS COTAS SE EXPRESAN EN MM
ACABADO SUPERFICIAL:
TOLERANCIAS:
LINEAL:
ANGULAR:

ACABADO:

REBARBAR Y ROMPER ARISTAS VIVAS

NO CAMBIE LA ESCALA

REVISIÓN

	NOMBRE	FIRMA	FECHA
DIBUJ.	Martín Ruiz Gutiérrez		
VERIF.			
APROB.			
FABR.			
CALID.			

TÍTULO:

Displays window

MATERIAL:

Transparent PETG

N.º DE DIBUJO

B3

A4

PESO:

ESCALA:2:1

HOJA 1 DE 1

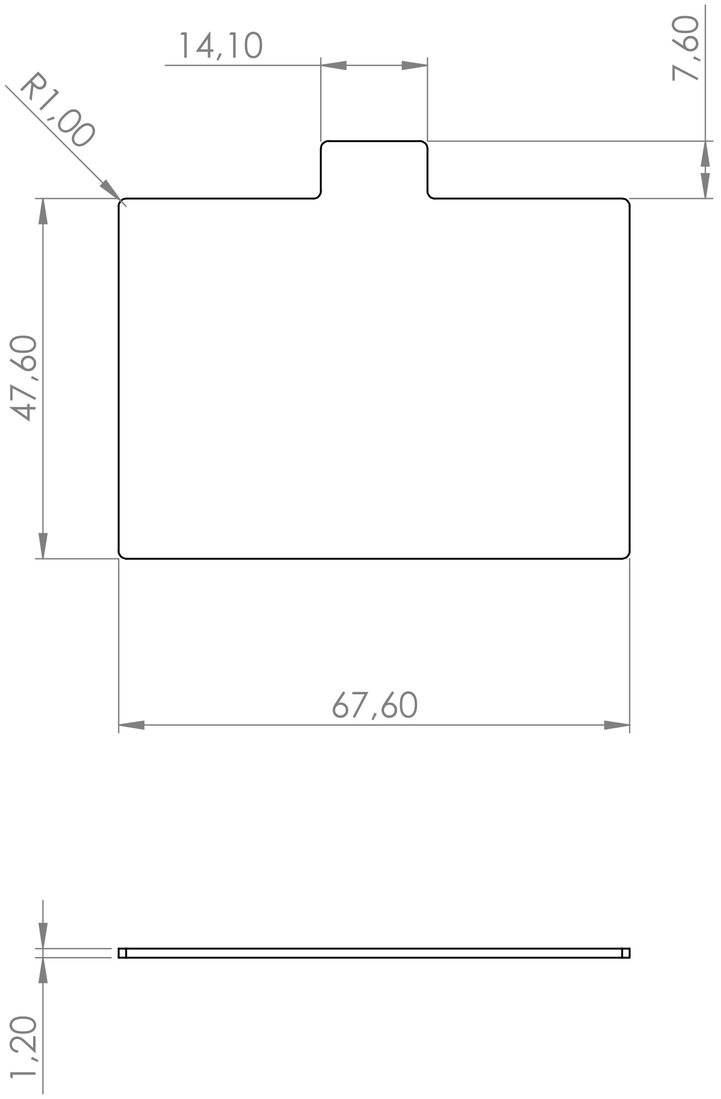

SI NO SE INDICA LO CONTRARIO:
LAS COTAS SE EXPRESAN EN MM
ACABADO SUPERFICIAL:
TOLERANCIAS:
LINEAL:
ANGULAR:

ACABADO:

REBARBAR Y ROMPER ARISTAS VIVAS

NO CAMBIE LA ESCALA

REVISIÓN

	NOMBRE	FIRMA	FECHA
DIBUJ.	Martín Ruiz Gutiérrez		
VERIF.			
APROB.			
FABR.			
CALID.			

TÍTULO:

LEDs window

MATERIAL:

Transparent PETG

N.º DE DIBUJO

B4

A4

PESO:

ESCALA:1:1

HOJA 1 DE 1

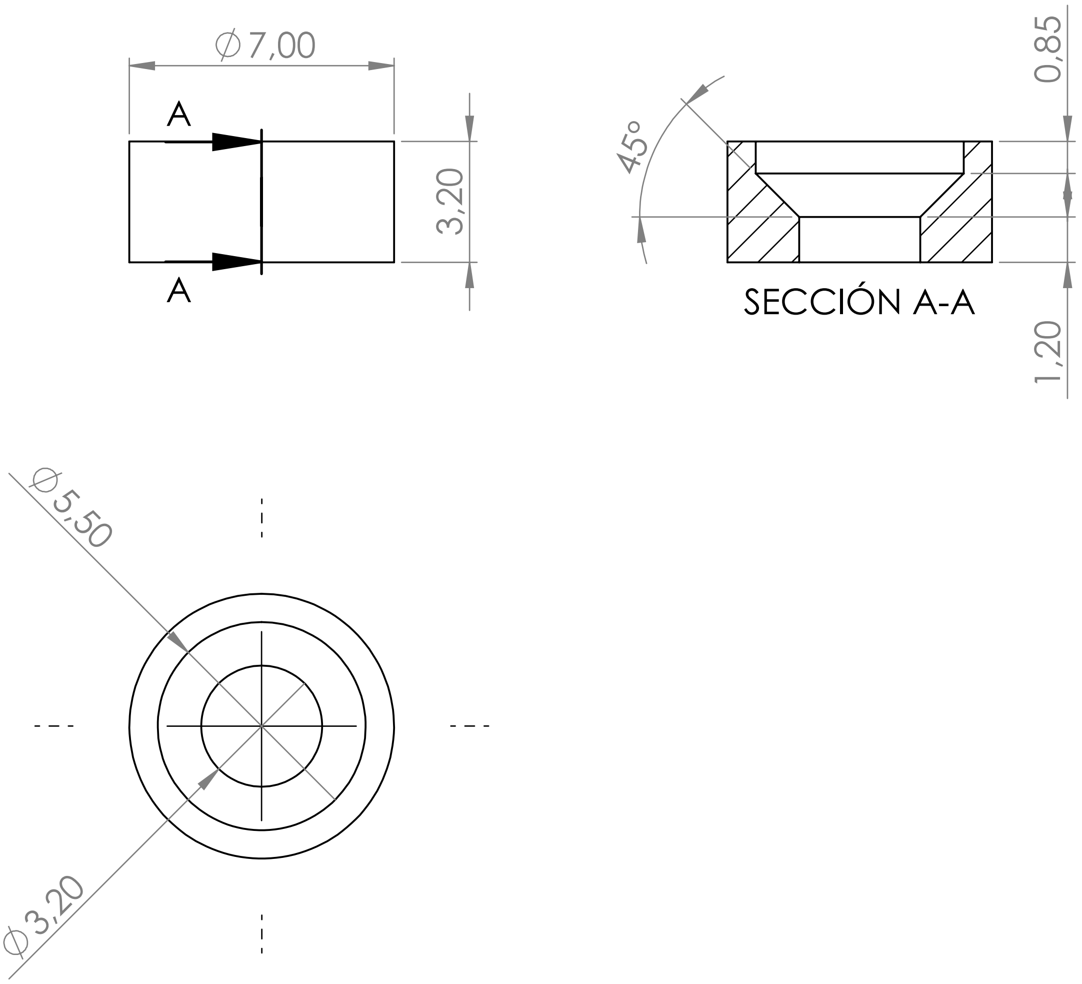

SI NO SE INDICA LO CONTRARIO:
LAS COTAS SE EXPRESAN EN MM
ACABADO SUPERFICIAL:
TOLERANCIAS:
LINEAL:
ANGULAR:

ACABADO:

REBARBAR Y ROMPER ARISTAS VIVAS

NO CAMBIE LA ESCALA

REVISIÓN

	NOMBRE	FIRMA	FECHA
DIBUJ.	Martín Ruiz Gutiérrez		
VERIF.			
APROB.			
FABR.			
CALID.			

TÍTULO:

M4 screw stand

MATERIAL:
TPU

N.º DE DIBUJO
B5

A4

PESO:

ESCALA:5:1

HOJA 1 DE 1

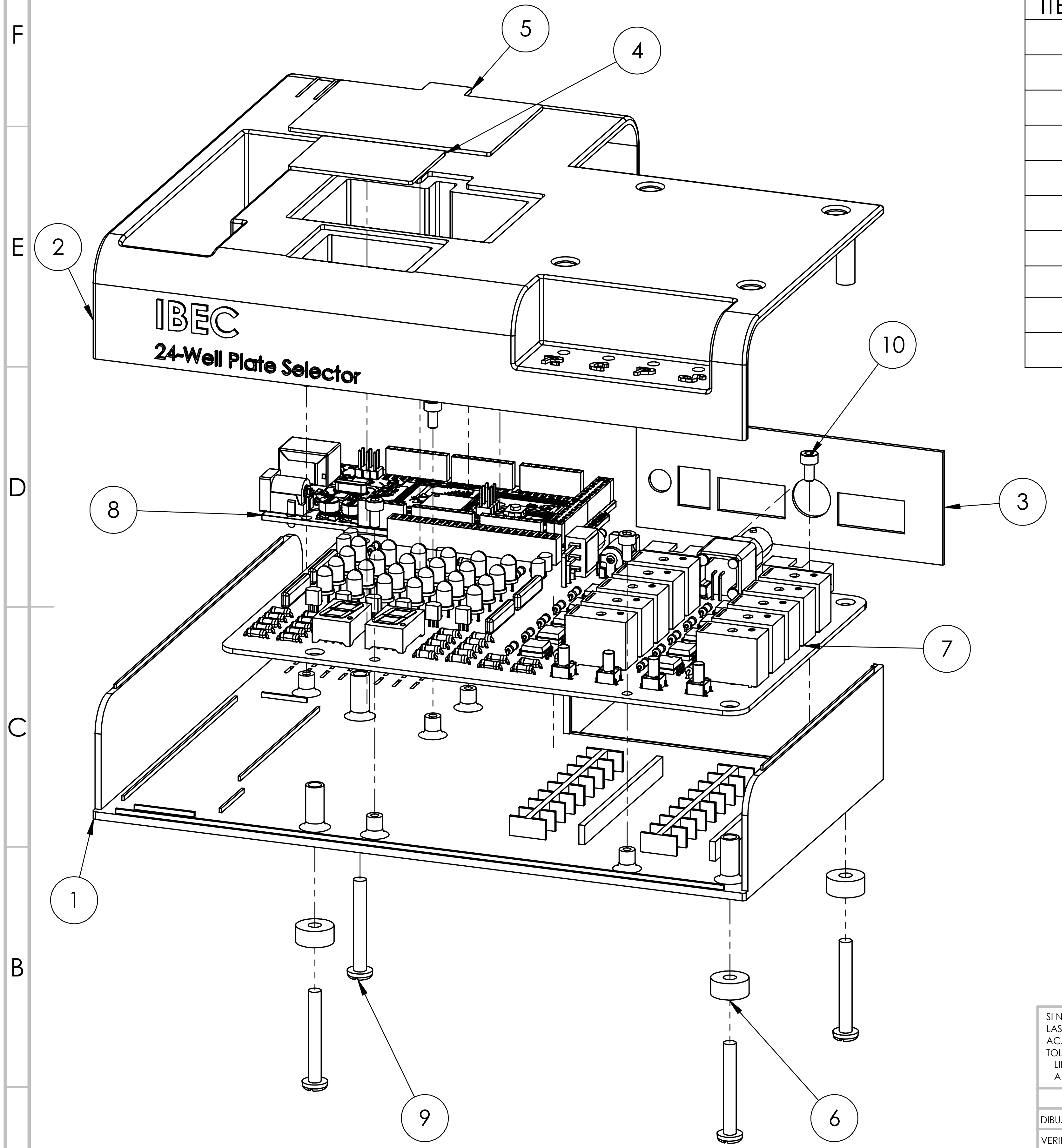

ITEM NO.	PART NUMBER	DESCRIPTION	QTY.
1	B0	Selector box base	1
2	B1	Selector box lid	1
3	B2	Selector box rear lid	1
4	B3	Displays window	1
5	B4	LEDs window	1
6	B5	M4 screw stand	4
7	-	Selector PCB	1
8	-	Arduino Mega 2560 Rev3	1
9	M4 x 30	M4 screw, 30 mm length	4
10	M3 x 6	M3 screw, 6 mm length	7

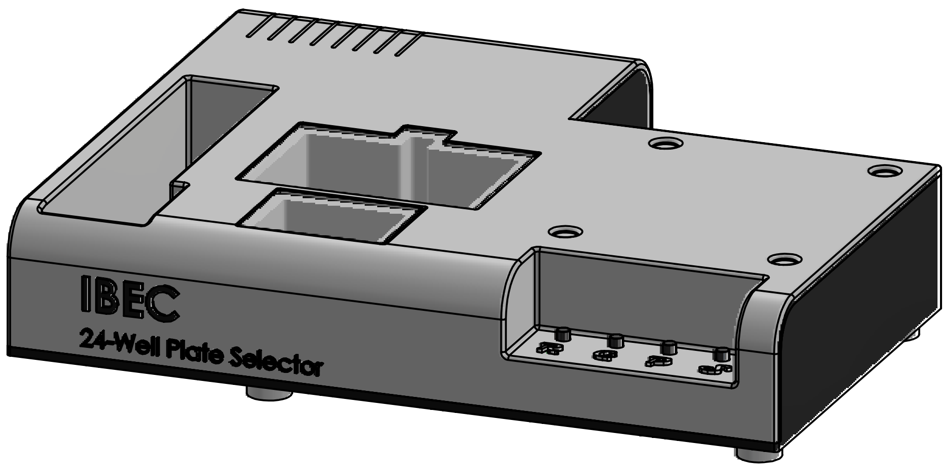

SI NO SE INDICA LO CONTRARIO:
LAS COTAS SE EXPRESAN EN MM
ACABADO SUPERFICIAL:
TOLERANCIAS:
LINEAL:
ANGULAR:

ACABADO:

REBARBAR Y ROMPER ARISTAS VIVAS

NO CAMBIE LA ESCALA

REVISIÓN

	NOMBRE	FIRMA	FECHA
DIBUJ.	Martín Ruiz Gutiérrez		
VERIF.			
APROB.			
FABR.			
CALID.			

MATERIAL:

PESO:

TÍTULO:

Selector assembly

N.º DE DIBUJO

B6

A3

ESCALA:1:5

HOJA 1 DE 1

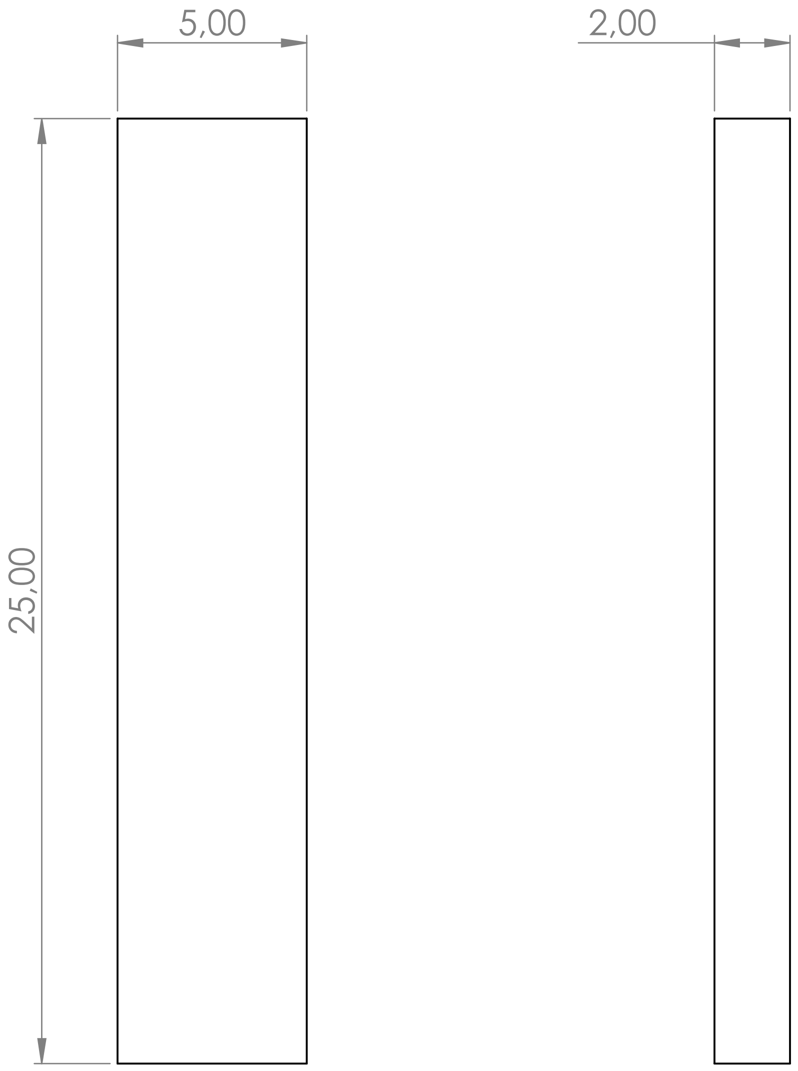

SI NO SE INDICA LO CONTRARIO:
LAS COTAS SE EXPRESAN EN MM
ACABADO SUPERFICIAL:
TOLERANCIAS:
LINEAL:
ANGULAR:

ACABADO:

REBARBAR Y ROMPER ARISTAS VIVAS

NO CAMBIE LA ESCALA

REVISIÓN

	NOMBRE	FIRMA	FECHA
DIBUJ.	Martín Ruiz Gutiérrez		
VERIF.			
APROB.			
FABR.			
CALID.			

TÍTULO:

Electrode

MATERIAL:
High purity graphite

N.º DE DIBUJO
C0

A4

PESO:

ESCALA:5:1

HOJA 1 DE 1

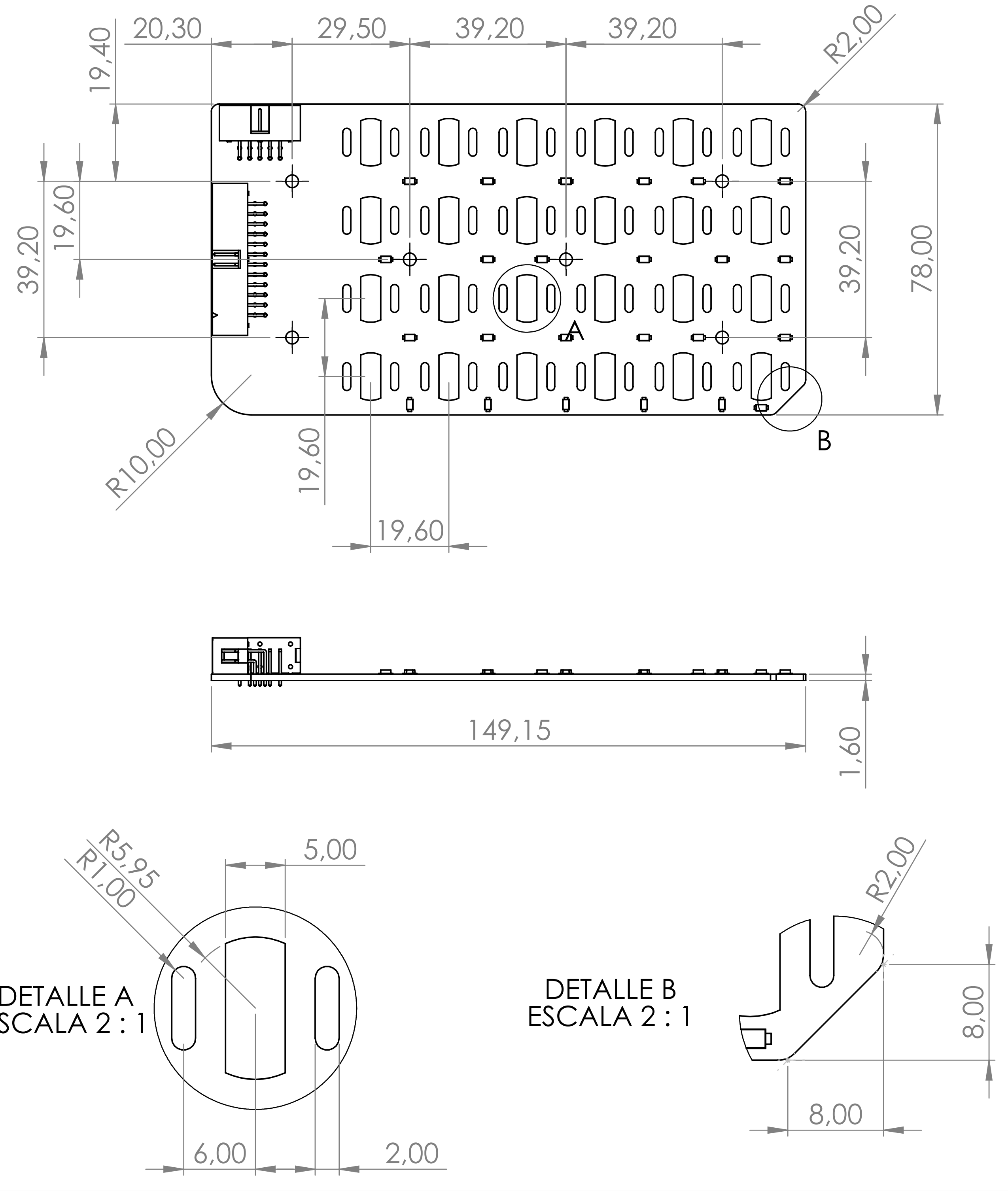

SI NO SE INDICA LO CONTRARIO:
LAS COTAS SE EXPRESAN EN MM
ACABADO SUPERFICIAL:
TOLERANCIAS:
LINEAL:
ANGULAR:

ACABADO:

REBARBAR Y ROMPER ARISTAS VIVAS

NO CAMBIE LA ESCALA

REVISIÓN

	NOMBRE	FIRMA	FECHA
DIBUJ.	Martín Ruiz Gutiérrez		
VERIF.			
APROB.			
FABR.			
CALID.			

MATERIAL:

PESO:

TÍTULO:

Stimulator PCB

N.º DE DIBUJO

C1

A4

ESCALA:1:1.5

HOJA 1 DE 1

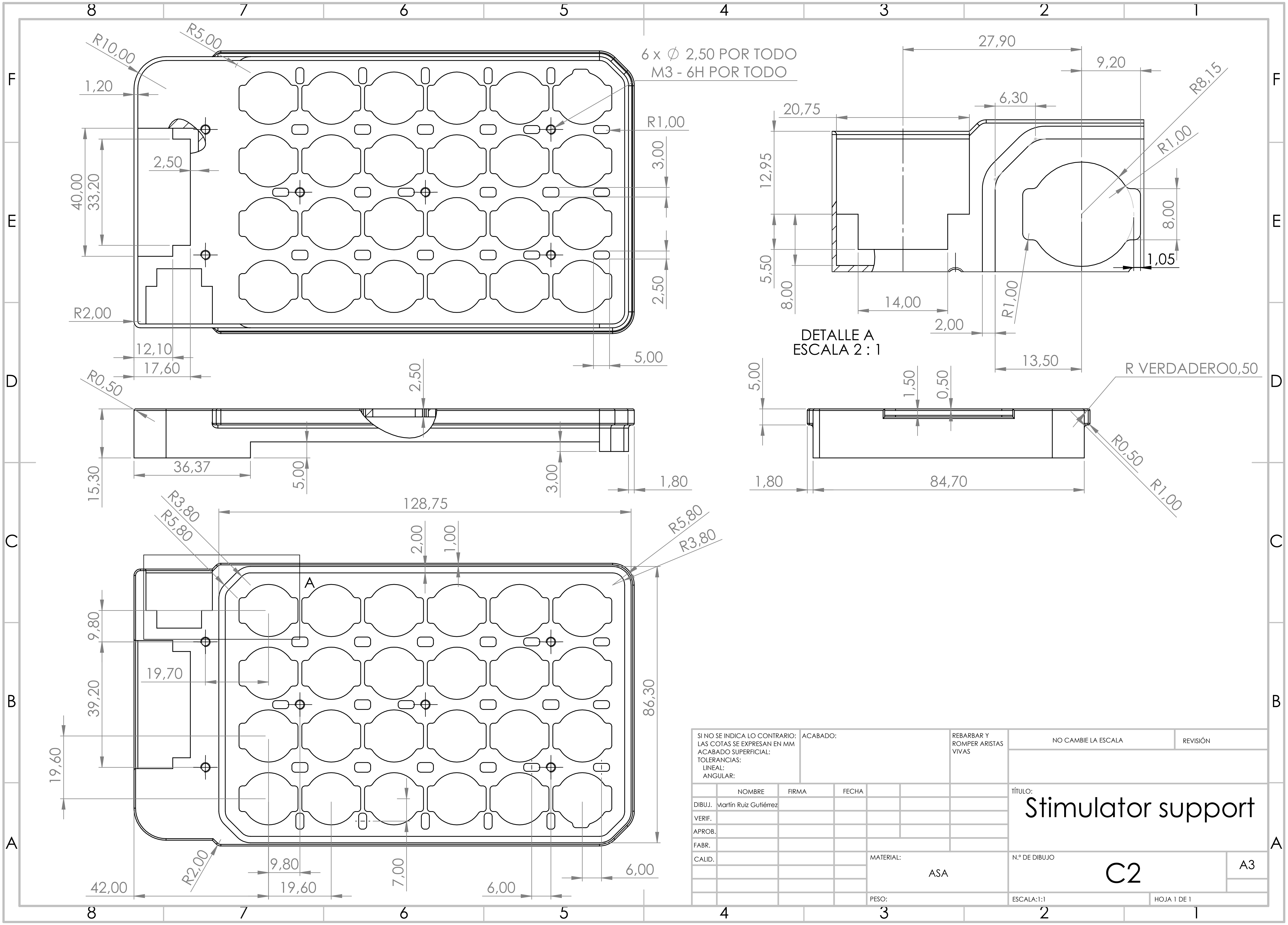

SI NO SE INDICA LO CONTRARIO: LAS COTAS SE EXPRESAN EN MM ACABADO SUPERFICIAL: TOLERANCIAS: LINEAL: ANGULAR:	ACABADO:	REBARBAR Y ROMPER ARISTAS VIVAS	NO CAMBIE LA ESCALA — REVISIÓN

	NOMBRE	FIRMA	FECHA
DIBUJ.	Martín Ruiz Gutiérrez		
VERIF.			
APROB.			
FABR.			
CALID.			

TÍTULO:

Stimulator support

MATERIAL: ASA

N.º DE DIBUJO: C2

A3

PESO:

ESCALA:1:1

HOJA 1 DE 1

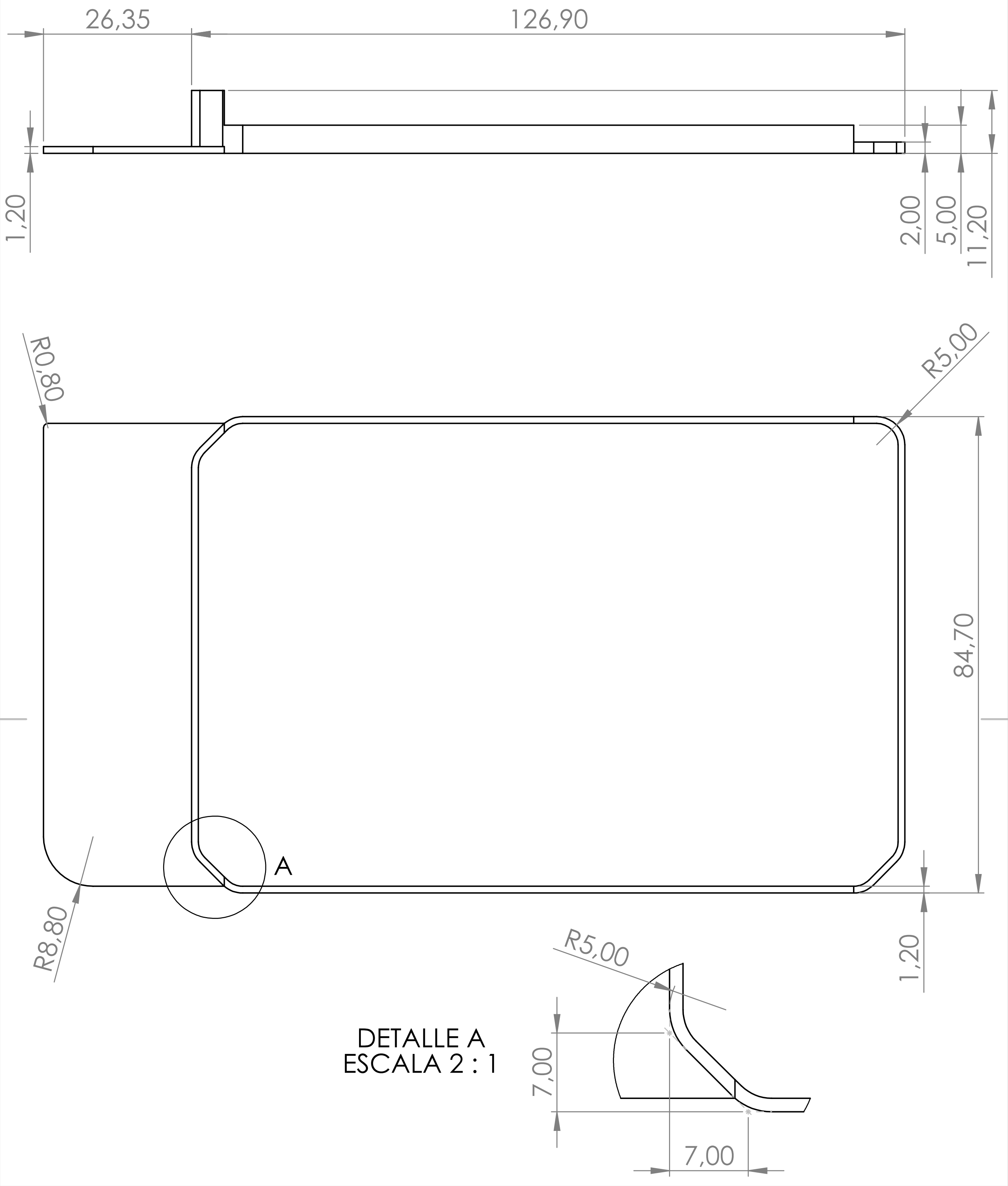

SI NO SE INDICA LO CONTRARIO:
LAS COTAS SE EXPRESAN EN MM
ACABADO SUPERFICIAL:
TOLERANCIAS:
LINEAL:
ANGULAR:

ACABADO:

REBARBAR Y ROMPER ARISTAS VIVAS

NO CAMBIE LA ESCALA

REVISIÓN

	NOMBRE	FIRMA	FECHA
DIBUJ.	Martín Ruiz Gutiérrez		
VERIF.			
APROB.			
FABR.			
CALID.			

TÍTULO:

Stimulator support base

MATERIAL:

PLA

N.º DE DIBUJO

C3

A4

PESO:

ESCALA:1:1

HOJA 1 DE 1

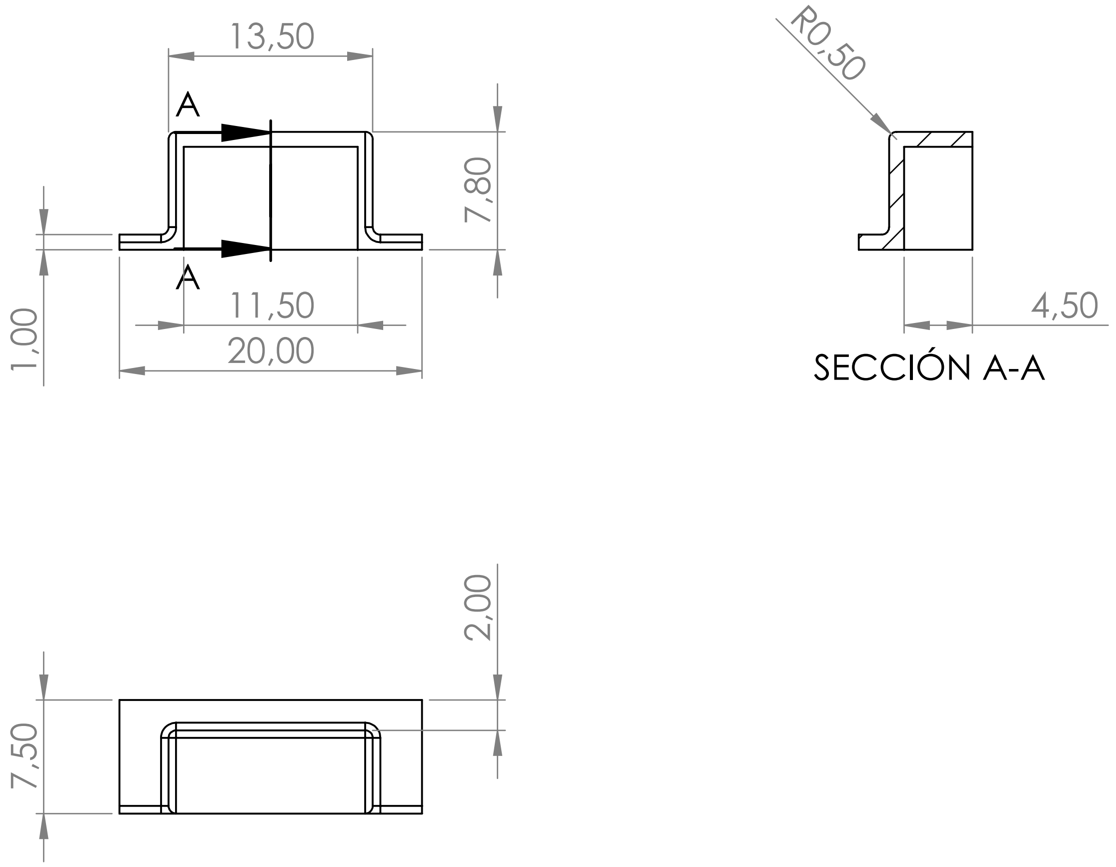

SI NO SE INDICA LO CONTRARIO:
LAS COTAS SE EXPRESAN EN MM
ACABADO SUPERFICIAL:
TOLERANCIAS:
LINEAL:
ANGULAR:

ACABADO:

REBARBAR Y ROMPER ARISTAS VIVAS

NO CAMBIE LA ESCALA

REVISIÓN

	NOMBRE	FIRMA	FECHA
DIBUJ.	Martín Ruiz Gutiérrez		
VERIF.			
APROB.			
FABR.			
CALID.			

TÍTULO:

Stimulator connector cover

MATERIAL: ASA

N.º DE DIBUJO: C4

A4

PESO:

ESCALA:2:1

HOJA 1 DE 1

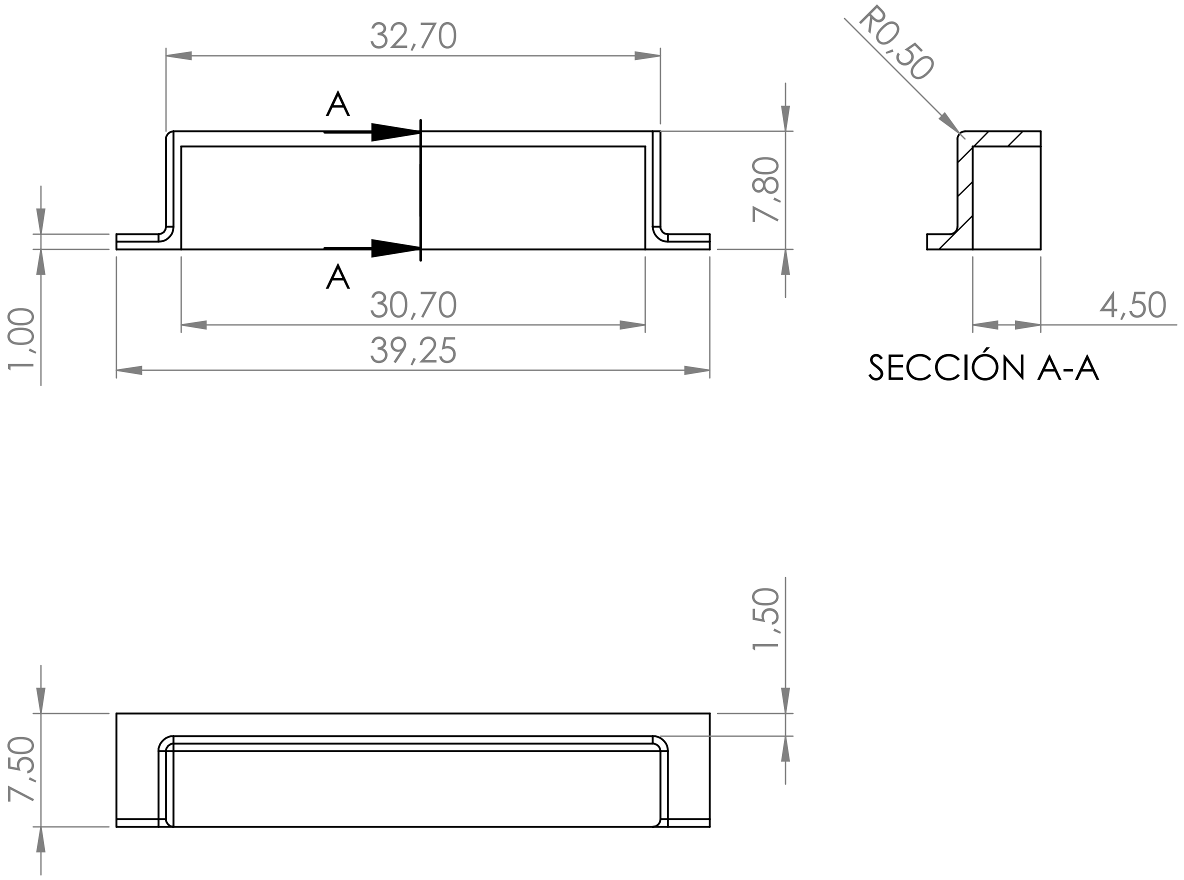

SI NO SE INDICA LO CONTRARIO:
LAS COTAS SE EXPRESAN EN MM
ACABADO SUPERFICIAL:
TOLERANCIAS:
LINEAL:
ANGULAR:

ACABADO:

REBARBAR Y ROMPER ARISTAS VIVAS

NO CAMBIE LA ESCALA

REVISIÓN

	NOMBRE	FIRMA	FECHA
DIBUJ.	Martín Ruiz Gutiérrez		
VERIF.			
APROB.			
FABR.			
CALID.			

TÍTULO:

Oscilloscope probes connector cover

MATERIAL:
ASA

N.º DE DIBUJO
C5

A4

PESO:

ESCALA:2:1

HOJA 1 DE 1

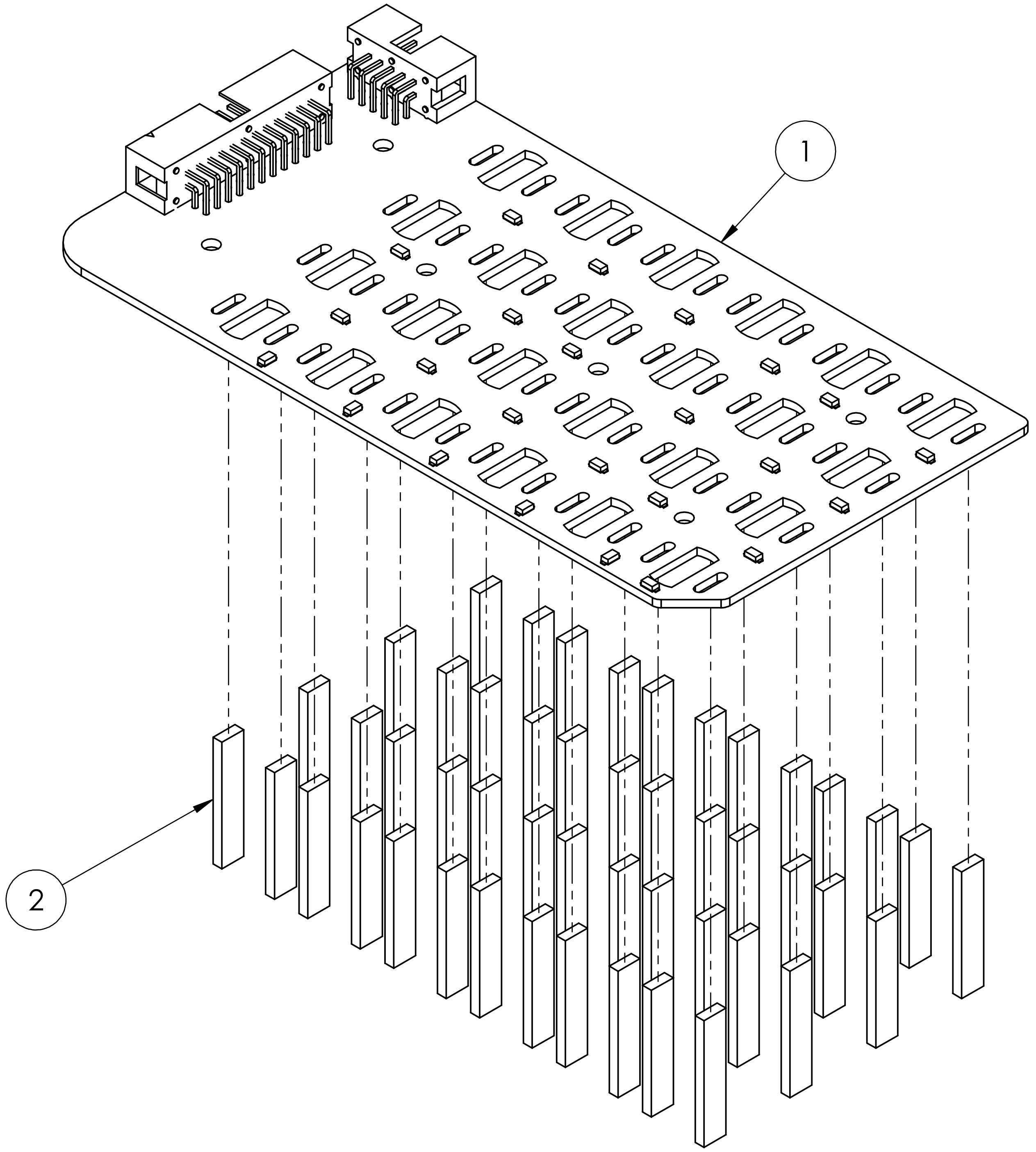

ITEM NO.	PART NUMBER	DESCRIPTION	QTY.
1	C1	Stimulator PCB	1
2	C0	Electrode	48

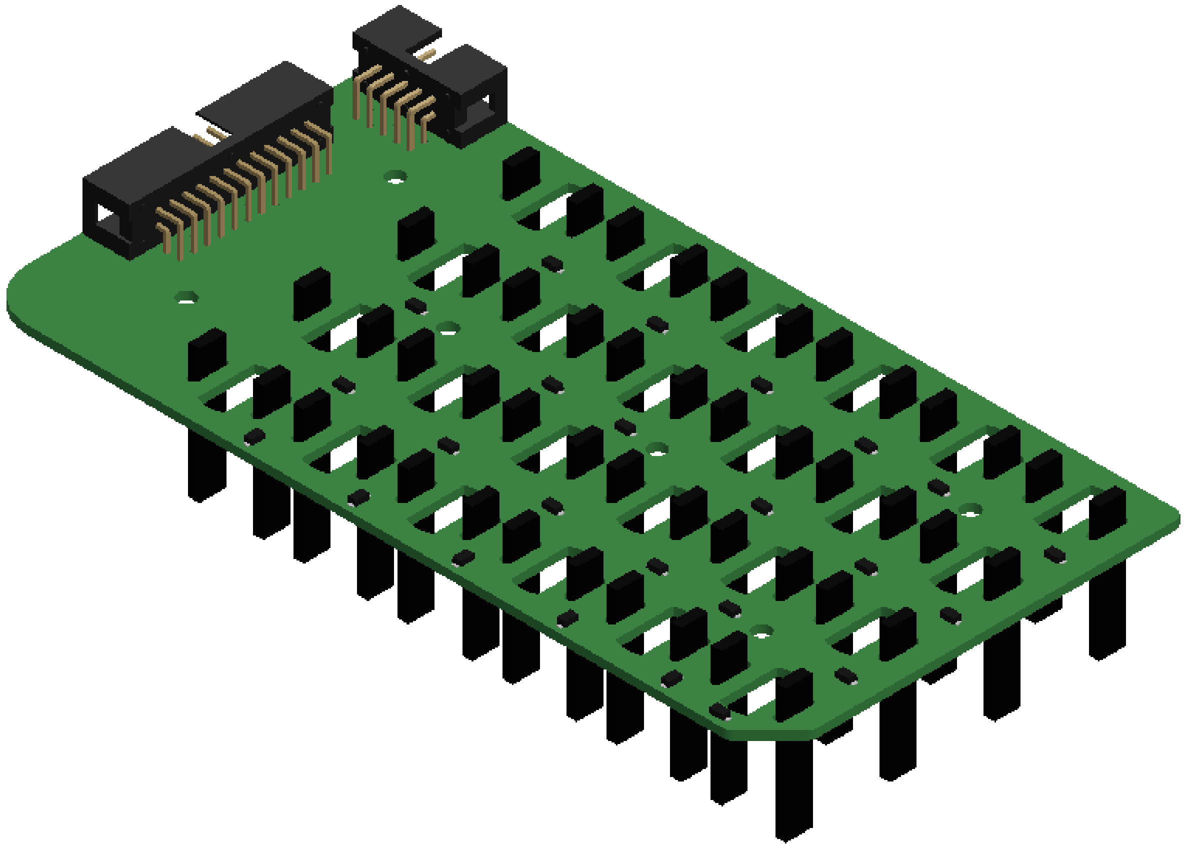

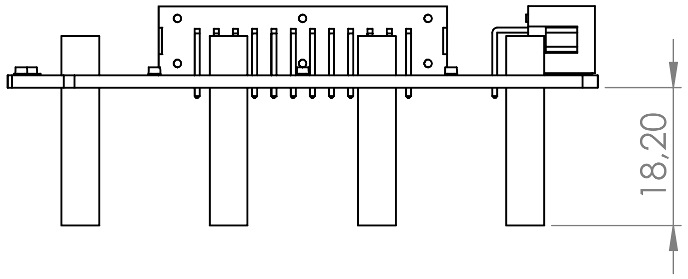

SI NO SE INDICA LO CONTRARIO:
LAS COTAS SE EXPRESAN EN MM
ACABADO SUPERFICIAL:
TOLERANCIAS:
LINEAL:
ANGULAR:

ACABADO:

REBARBAR Y ROMPER ARISTAS VIVAS

NO CAMBIE LA ESCALA

REVISIÓN

	NOMBRE	FIRMA	FECHA
DIBUJ.	Martín Ruiz Gutiérrez		
VERIF.			
APROB.			
FABR.			
CALID.			

MATERIAL:

PESO:

TÍTULO:

Stimulator PCB assembly

N.º DE DIBUJO

C6

A3

ESCALA:1:1

HOJA 1 DE 1

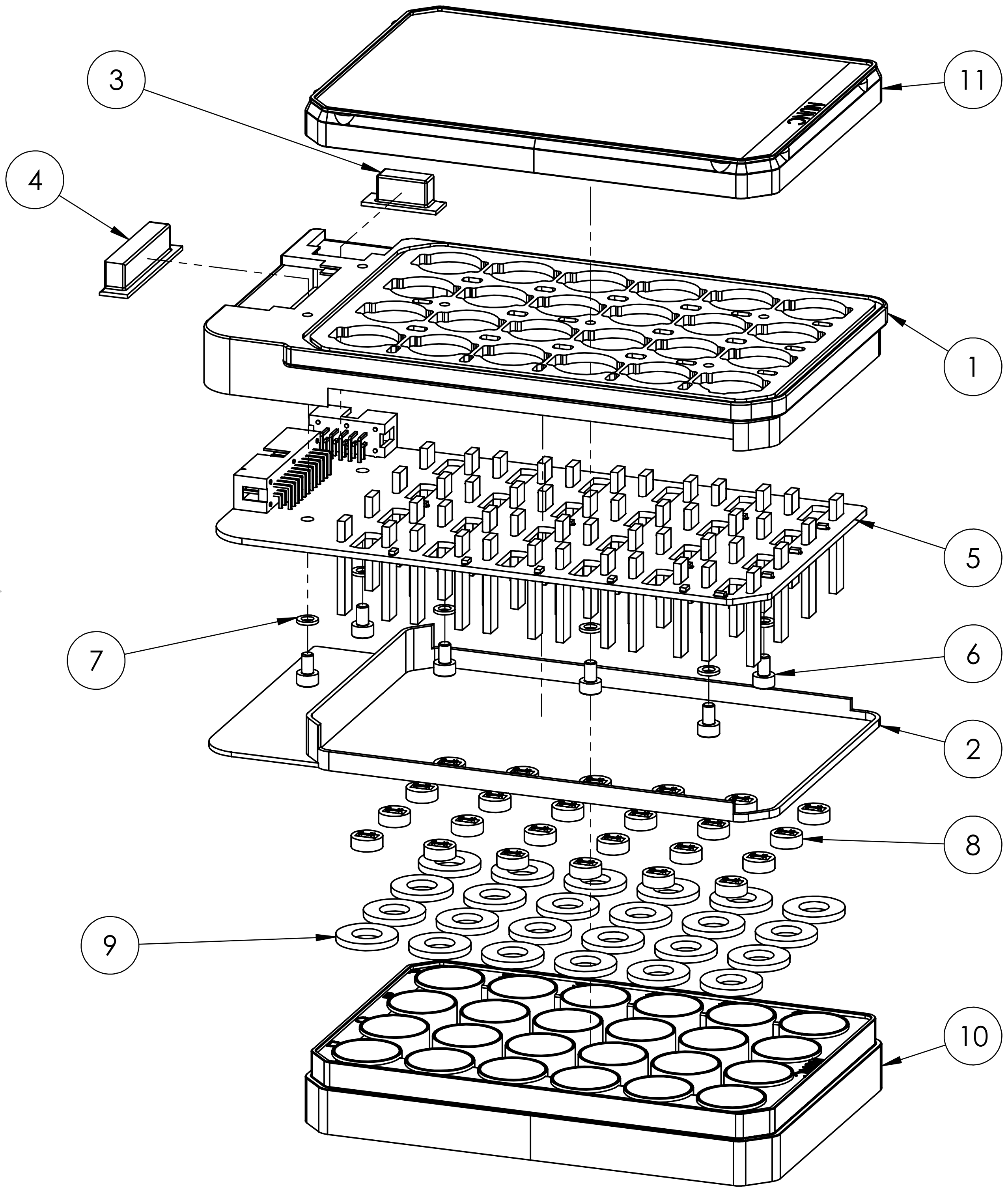

ITEM NO.	PART NUMBER	DESCRIPTION	QTY.
1	C2	Stimulator support	1
2	C3	Stimulator support base	1
3	C4	Stimulator connector cover	1
4	C5	Oscilloscope probes connector cover	1
5	C6	Stimulator PCB assembly	1
6	ISO 4762 M3 x 5 - 5C	M3 screw, 6 mm long, SS	6
7	-	M3 washer	6
8	-	PDMS mould with sample	24
9	-	PDMS ring	24
10	-	Thermofisher NUNC 24 Well Plate	1
11	-	Thermofisher NUNC 24 Well Plate Lid	1

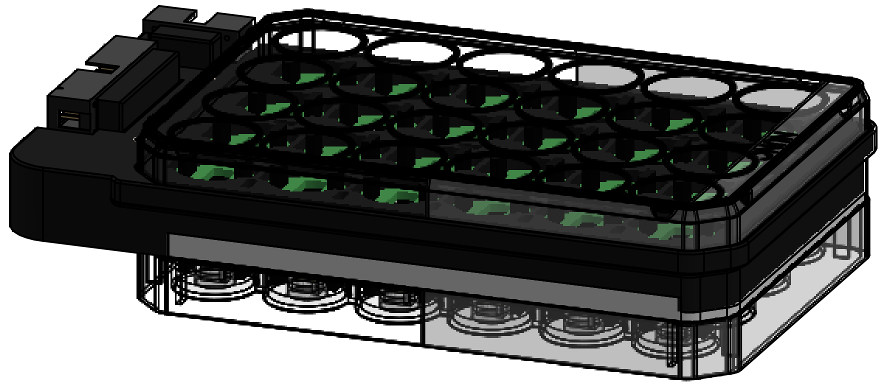

SI NO SE INDICA LO CONTRARIO: LAS COTAS SE EXPRESAN EN MM ACABADO SUPERFICIAL: TOLERANCIAS: LINEAL: ANGULAR:

ACABADO:

REBARBAR Y ROMPER ARISTAS VIVAS

NO CAMBIE LA ESCALA

REVISIÓN

	NOMBRE	FIRMA	FECHA
DIBUJ.	Martín Ruiz Gutiérrez		
VERIF.			
APROB.			
FABR.			
CALID.			

TÍTULO: Stimulator assembly

MATERIAL:

N.º DE DIBUJO: C7

A3

PESO:

ESCALA:1:1.5

HOJA 1 DE 1

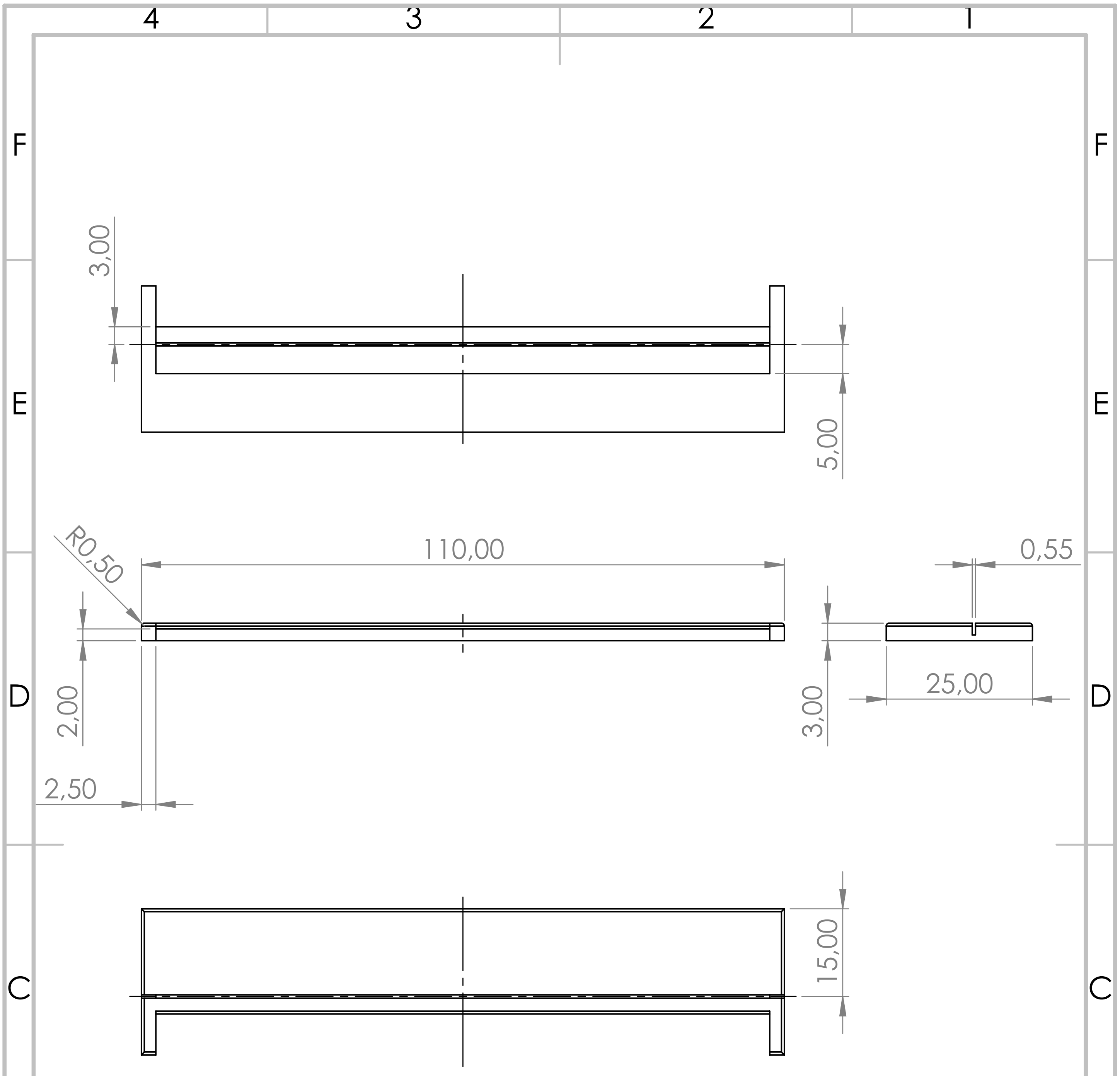

SI NO SE INDICA LO CONTRARIO:
LAS COTAS SE EXPRESAN EN MM
ACABADO SUPERFICIAL:
TOLERANCIAS:
LINEAL:
ANGULAR:

ACABADO:

REBARBAR Y ROMPER ARISTAS VIVAS

NO CAMBIE LA ESCALA

REVISIÓN

	NOMBRE	FIRMA	FECHA
DIBUJ.	Martín RUiz Gutiérrez		
VERIF.			
APROB.			
FABR.			
CALID.			

TÍTULO:

Longitudinal electrode cutting support

MATERIAL: PLA

N.º DE DIBUJO: D0

A4

PESO:

ESCALA:1:1

HOJA 1 DE 1

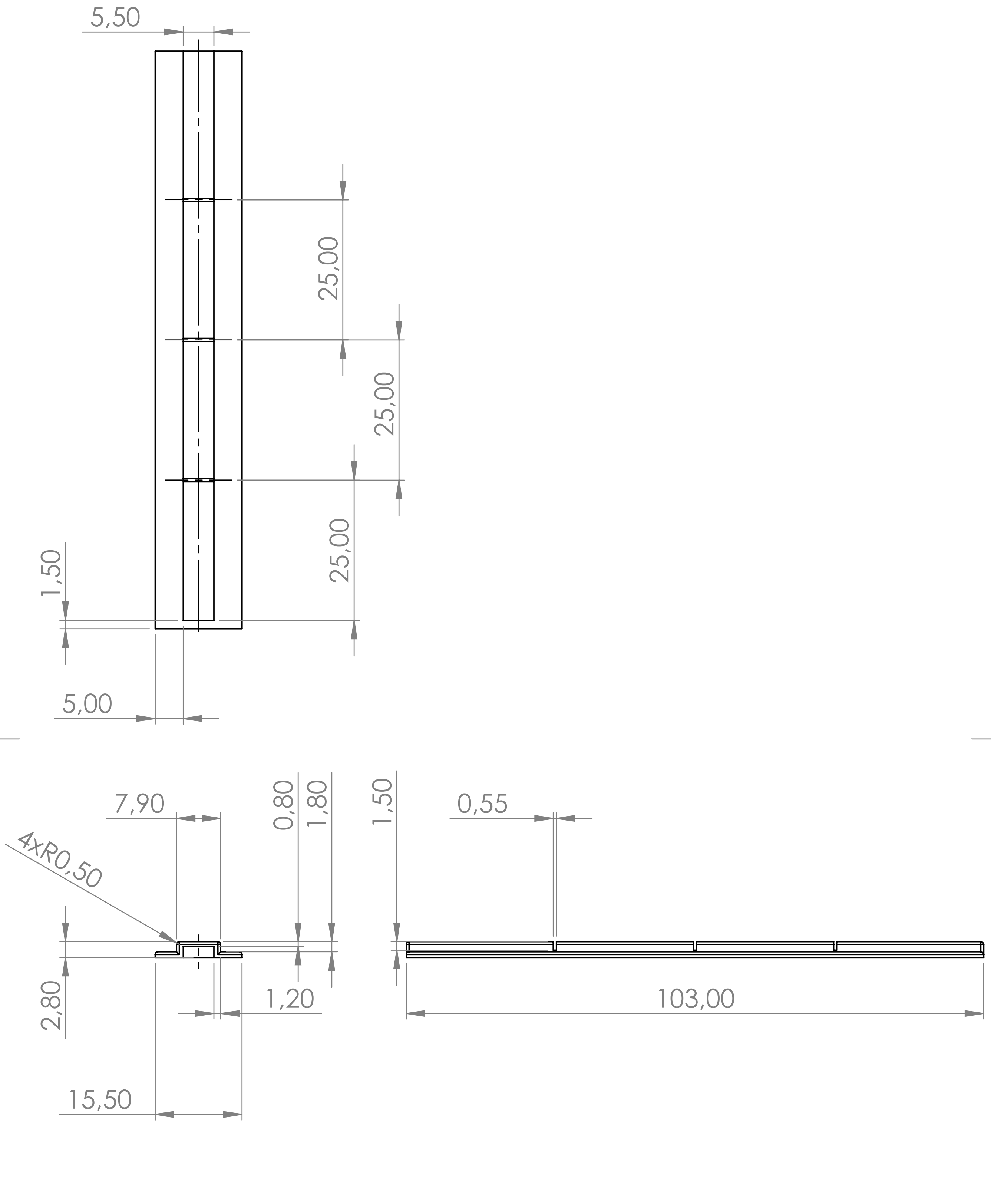

SI NO SE INDICA LO CONTRARIO:
LAS COTAS SE EXPRESAN EN MM
ACABADO SUPERFICIAL:
TOLERANCIAS:
LINEAL:
ANGULAR:

ACABADO:

REBARBAR Y ROMPER ARISTAS VIVAS

NO CAMBIE LA ESCALA

REVISIÓN

	NOMBRE	FIRMA	FECHA
DIBUJ.	Martín Ruiz Gutiérrez		
VERIF.			
APROB.			
FABR.			
CALID.			

TÍTULO:

Transversal electrode cutting support

MATERIAL: PLA

N.º DE DIBUJO: D1

A4

PESO:

ESCALA:1:1

HOJA 1 DE 1

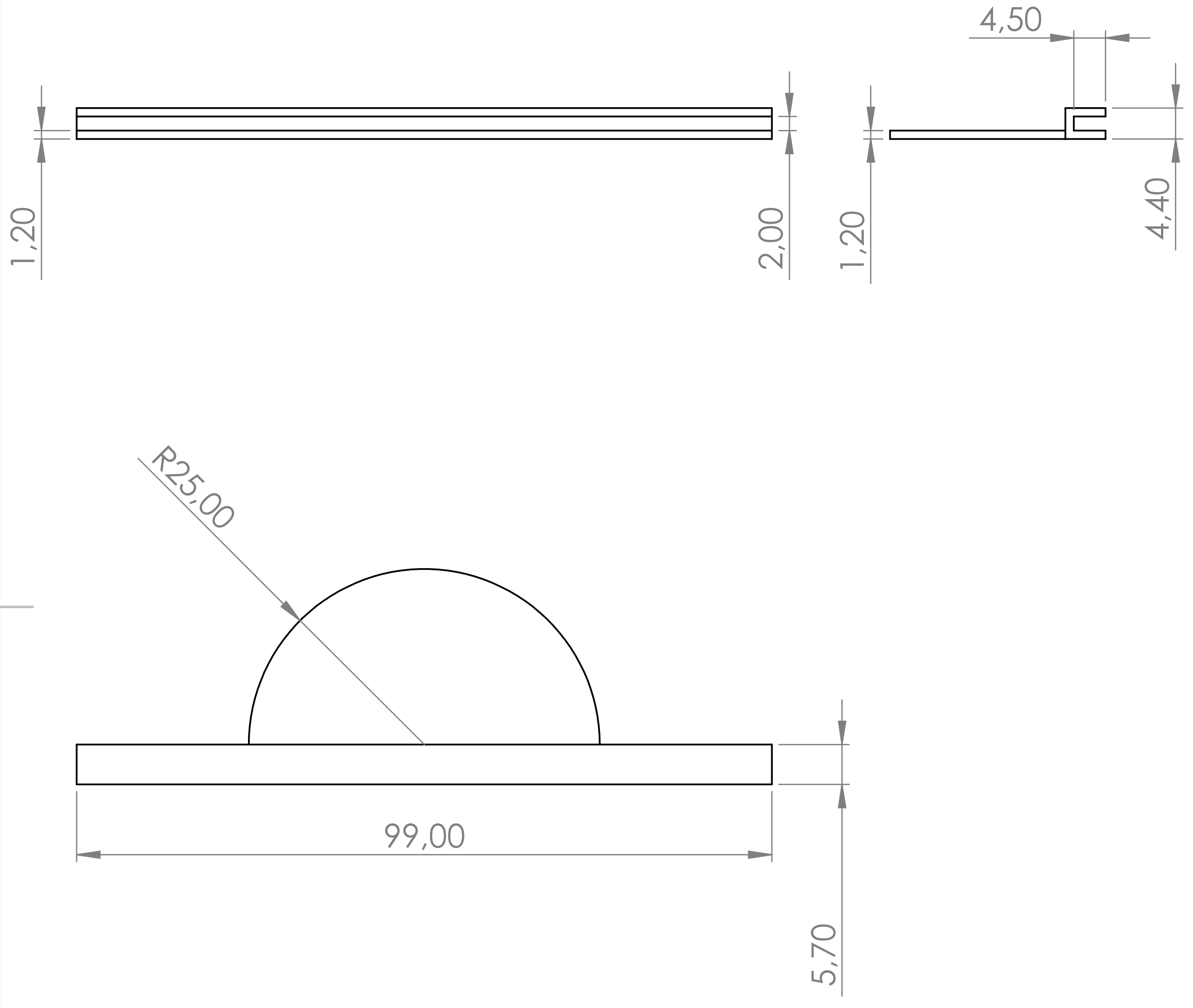

SI NO SE INDICA LO CONTRARIO: LAS COTAS SE EXPRESAN EN MM ACABADO SUPERFICIAL: TOLERANCIAS: LINEAL: ANGULAR:	ACABADO:	REBARBAR Y ROMPER ARISTAS VIVAS	NO CAMBIE LA ESCALA	REVISIÓN

	NOMBRE	FIRMA	FECHA
DIBUJ.	Martín Ruiz Gutiérrez		
VERIF.			
APROB.			
FABR.			
CALID.			

TÍTULO:

Electrode cutting lever

MATERIAL: PLA

N.º DE DIBUJO: D2

A4

PESO:

ESCALA:1:1

HOJA 1 DE 1

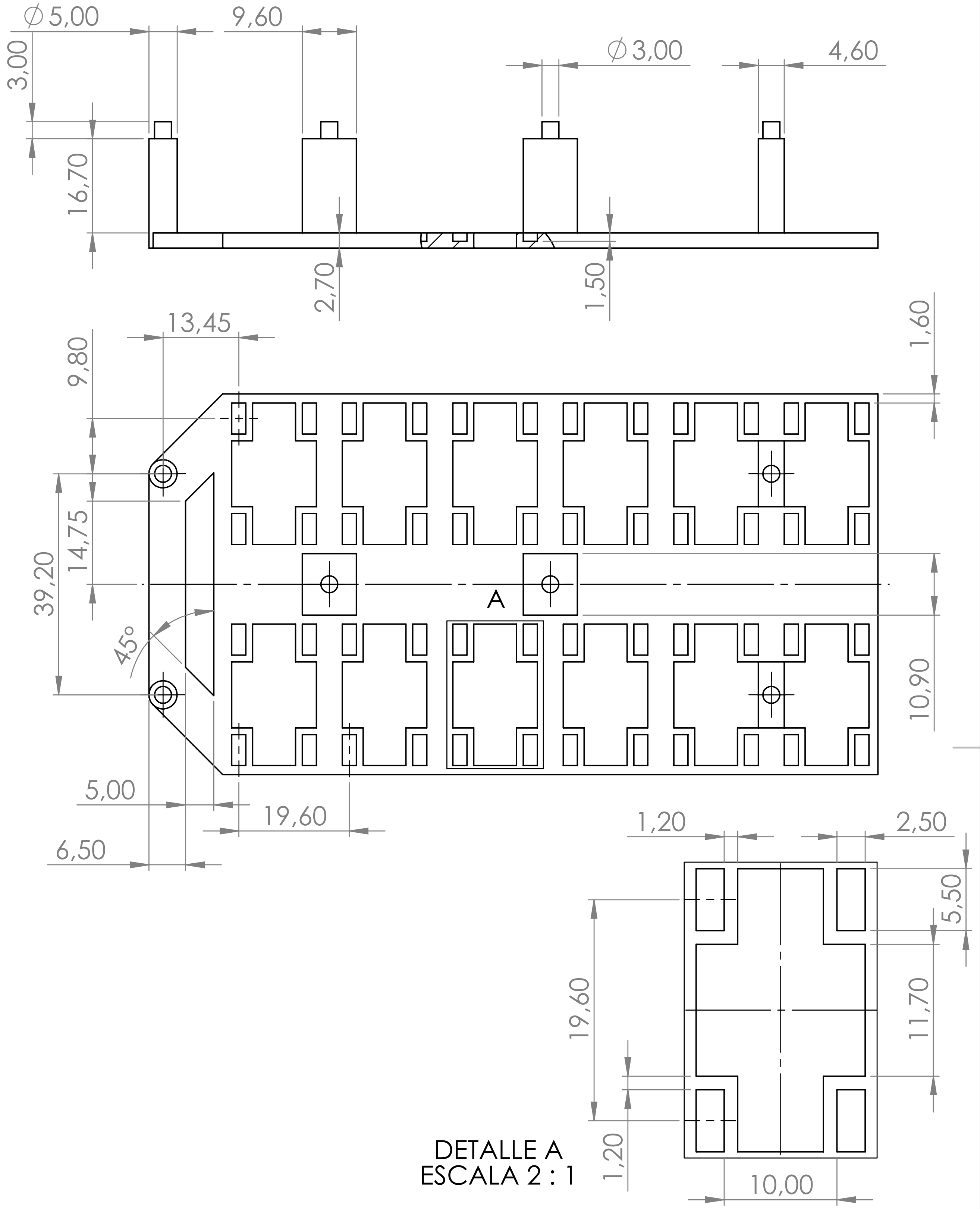

DETALLE A
ESCALA 2 : 1

SI NO SE INDICA LO CONTRARIO:
LAS COTAS SE EXPRESAN EN MM
ACABADO SUPERFICIAL:
TOLERANCIAS:
LINEAL:
ANGULAR:

ACABADO:

REBARBAR Y ROMPER ARISTAS VIVAS

NO CAMBIE LA ESCALA

REVISIÓN

	NOMBRE	FIRMA	FECHA
DIBUJ.			
VERIF.			
APROB.			
FABR.			
CALID.			

TÍTULO:

Electrode mounting guide

MATERIAL:

N.º DE DIBUJO

D3

A4

PESO:

ESCALA:1:1

HOJA 1 DE 1
